# Phytochrome A Regulates Carbon Flux in Dark Grown Tomato Seedlings

**DOI:** 10.3389/fpls.2019.00152

**Published:** 2019-02-27

**Authors:** Keisha D. Carlson, Sneha Bhogale, Drew Anderson, Lars Tomanek, Andreas Madlung

**Affiliations:** ^1^Department of Biology, University of Puget Sound, Tacoma, WA, United States; ^2^Department of Biology, California Polytechnic State University, San Luis Obispo, CA, United States

**Keywords:** phytochrome, primary metabolism, tricarboxylic acid (TCA) cycle, glycolysis, storage proteins, beta oxidation, skotomorphogenesis, photomorphogenesis

## Abstract

Phytochromes comprise a small family of photoreceptors with which plants gather environmental information that they use to make developmental decisions, from germination to photomorphogenesis to fruit development. Most phytochromes are activated by red light and de-activated by far-red light, but phytochrome A (phyA) is responsive to both and plays an important role during the well-studied transition of seedlings from dark to light growth. The role of phytochromes during skotomorphogenesis (dark development) prior to reaching light, however, has received considerably less attention although previous studies have suggested that phytochrome must play a role even in the dark. We profiled proteomic and transcriptomic seedling responses in tomato during the transition from dark to light growth and found that phyA participates in the regulation of carbon flux through major primary metabolic pathways, such as glycolysis, beta-oxidation, and the tricarboxylic acid (TCA) cycle. Additionally, phyA is involved in the attenuation of root growth soon after reaching light, possibly via control of sucrose allocation throughout the seedling by fine-tuning the expression levels of several sucrose transporters of the *SWEET* gene family even before the seedling reaches the light. Presumably, by participating in the control of major metabolic pathways, phyA sets the stage for photomorphogenesis for the dark grown seedling in anticipation of light.

## Introduction

Plants use light not only as a source of energy but also as an environmental cue that can trigger developmental changes. As sessile organisms, sensing the surrounding environment and coordinating their development in response is necessary for plant survival. Photoreceptors, such as phytochromes, cryptochromes, and phototropins, sense light conditions and direct cellular and developmental responses (Demarsy and Fankhauser, 2009; Chen and Chory, 2011; Chaves et al., 2011).

Phytochromes, which specifically perceive the ratio of red light (R) to far red light (FR), play a well-studied role in photomorphogenesis by coordinating signal transduction pathways leading to changes in gene expression and eventually to de-etiolation (Weller et al., 2000; Tepperman et al., 2004, 2006; Leivar et al., 2009). Key to the transition from dark to light growth is the production of photosynthetic pigments that allow the plant to go from reliance on stored energy within the seed to an autotrophic life-style in the light. During dark growth (skotomorphogenesis), the plant uses energy stores to achieve maximal axis elongation while limiting lateral growth and pigment production. Upon sensing light, the plant slows its growth rate and begins producing secondary metabolites including light-harvesting pigments that allow the plant to start photosynthesizing (von Arnim and Deng, 1996; Chen et al., 2004). There is some evidence phytochromes also lead to the down-regulation of respiration on light exposure (Igamberdiev et al., 2014).

In Arabidopsis, where the phytochrome gene family consists of five members (*PHYA-E*), it has been suggested that light-activated phyB may play a role in the seed before germination, possibly preconditioning the developing seedling for photomorphogenesis while still growing in the dark (Mazzella et al., 2005). Exactly how plants coordinate optimal growth in the dark in anticipation of future exposure to light, and how they shift from using exclusively stored energy sources to producing energy via light capture is, however, still largely unknown.

To begin to understand to what degree, if any, phyA affects skotomorphogenesis before and during the transition to photomorphogenesis, we profiled the transcriptomes and proteomes of developing wild-type (WT) and *phyA* tomato seedlings both before and after their first exposure to light. We report here that phyA in tomato plays an important role in the coordination of carbon flux in primary metabolism and energy provisioning in seedlings both during skotomorphogenesis and immediately after the transition to photomorphogenesis.

## Results

**FIGURE 1:**
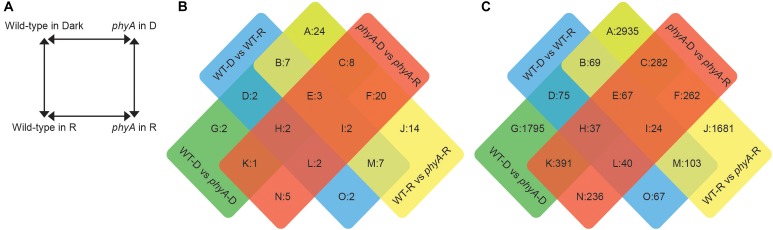
Differentially expressed transcripts and proteins between *phyA* mutants and WT in the dark and after exposure to R. **(A)** Experimental design: Comparisons to determine differential expression were made between four genotype/condition groups. Significantly differently expressed proteins **(B)** and transcripts **(C)** were assigned to a sector based on comparisons in which they were differentially expressed.

To determine the molecular role of phyA during skotomorphogenesis and seedling transition to light and to compare the tomato photomorphogenic response with that of the well-studied model plant Arabidopsis (Tepperman et al., 2006), we profiled the proteomes and transcriptomes of the tomato *phyA* mutant and WT seedlings under the same experimental conditions as described for published work in Arabidopsis (Tepperman et al., 2006). We made four comparisons to identify differentially expressed (DE) proteins and mRNA transcripts involved in the phyA-mediated R response: we compared *phyA* to WT in the dark, *phyA* to WT after 60 min of R, dark-grown WT to WT after 60 min of R, and dark-grown *phyA* to *phyA* after 60 min of R (Figure 1A). Using co-expression analysis, we found gene modules with co-expressed transcripts across the four sample groups (WT in the dark and in R, *phyA* in the dark and in R), and built co-expression networks. We then used gene ontology (GO) enrichment analysis on both the DE proteins and transcripts and the co-expression networks to better characterize the functions of proteins and transcripts regulated by phyA.

Across the four comparisons, proteomics analysis identified 204 (101 non-redundant) significantly differentially expressed proteins (DEPs) (Figure 1B and Supplementary Table S1). Of these significant DEPs, there were 72 unique gene IDs. Gene IDs were duplicated in the protein data because a given protein with a specific gene ID could be represented on the 2D gel multiple times as different proteolytic forms, isoforms or with different post-translational modifications (Gruis et al., 2002; Stephens et al., 2010; Fields et al., 2012), and indeed we saw significant differential expression of differently modified versions of the same protein, or different spots with the same gene ID, in the same comparison (Supplementary Table S1). The parallel RNA-seq analysis identified 12,895 (8,064 non-redundant) significantly differentially expressed transcripts (DETs) among the four comparisons (Figure 1C and Supplementary Table S2). Our biological replicates clustered cleanly in a principal components analysis (PCA, Supplementary Figure S1). The most abundant DEPs and DETs were those that were found in the WT to *phyA* mutant comparisons both in the dark and in R (sector A of Figure 1B,C), suggesting that the most striking differences are between genotypes and not between light conditions and that many of the differences between WT and *phyA* already exist before exposure to light.

To validate the RNA-seq results we selected five of DE genes and tested them with qPCR. We found relatively good agreement between qPCR and RNA-seq data in the patterns of the responses in RNA-seq and qPCR (Supplementary Figure S2). To further validate our RNA-seq results, we compared transcripts that we identified to be significantly induced or repressed by 60 min of R in WT tomato to those identified in the same conditions in *Arabidopsis thaliana* using microarrays (Tepperman et al., 2006). We found an overlap of 55 genes, 52 of which were regulated in the same direction, either repressed or induced in light (Supplementary Table S3). Using a permutation test, we determined that an overlap of 55 genes between 848 DETs from *A. thaliana* with tomato reciprocal best BLAST hit orthologs (Tepperman et al., 2006) and 482 DETs from WT dark versus WT in R from tomato (this study) is significantly more than one would expect by chance (*p* < 0.0001). The shared genes that are induced by R include the phy responsive transcription factor HY5, circadian clock transcription factors RVE1, RVE7, REV8, and LHY1, HSP70, β-amylases, and several photosynthesis genes. The shared genes repressed by R include circadian clock genes RAV1, a transcription factor, which may be a negative growth regulator (Hu et al., 2004) and GA5, which is important for gibberellin synthesis (Xu et al., 1995) along with two brassinosteroid responsive transcription factors: BEE2, known to positively regulate shade avoidance (Cifuentes-Esquivel et al., 2013) and BZS1, which suppresses photomorphogenesis in the dark (Fan et al., 2012) (Supplementary Table S3). Next, we compared genes found to be either induced or repressed by phyA in the study by Tepperman et al. (2006) to genes found to be DE in *phyA* mutants in our data. We noticed that of 195 *A. thaliana* genes with tomato orthologs found to be induced or repressed by phyA in 60 min R (Tepperman et al., 2006), 126 were found to be DE in at least one comparison including the *phyA* mutant, so they could also be said to be regulated by phyA although effects of *phyA* and R varied between species (Supplementary Table S4). Although our data largely agree with the work done in *A. thaliana* validating our approach, our analysis shows that there are also distinct differences between phyA’s role in the two species. For example, some genes that were upregulated by phyA in Arabidopsis were downregulated by phyA in tomato, and vice versa, while other genes did not appear to be affected by the mutation in phyA (Supplementary Table S4).

### Primary Metabolism Is Regulated by phyA During Seedling Growth in the Dark

**FIGURE 2:**
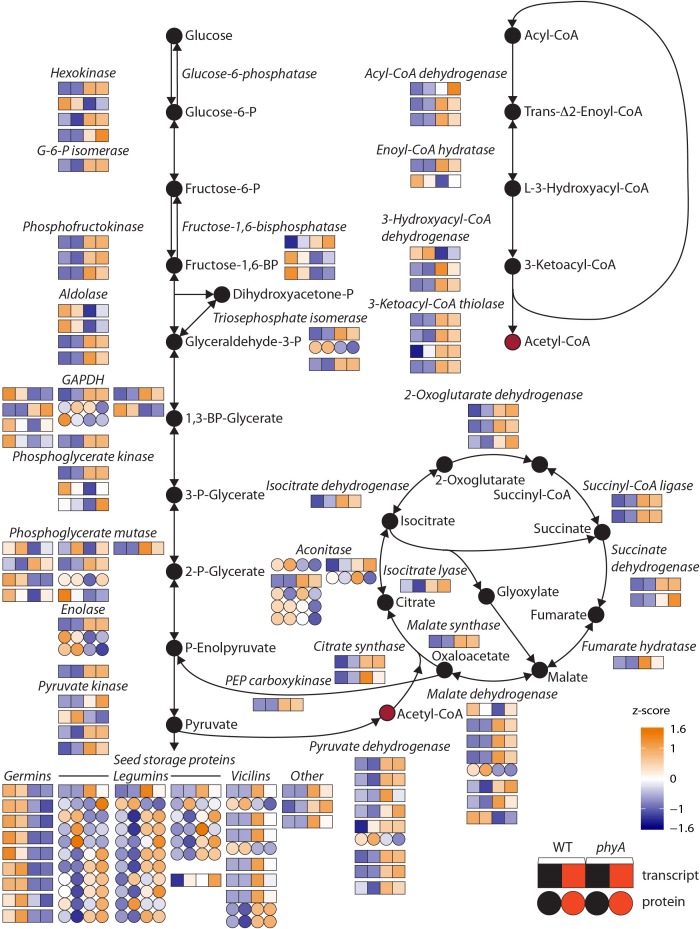
Primary metabolism pathway genes are differentially expressed at the transcript and/or protein level in *phyA* mutants compared to WT in the dark and after exposure to R. Normalized read counts [differentially expressed transcripts, (DETs), squares] or normalized spot volumes [differentially expressed proteins (DEPs), circles] were *Z*-score normalized to a color scale where white represents average expression across genotype/conditions. Glycolysis, the tricarboxylic acid cycle, the glyoxylate cycle, and beta-oxidation pathways are represented. For each enzyme, multiple gene IDs are annotated with that function. Genes are shown in order from top down: lowest chromosome number and coordinates to highest chromosome number and coordinates. If both the transcript and protein of the same gene ID are differentially expressed (DE), this is represented by connected circles and squares. If more than one protein spot was DE and identified as the same gene, this is represented by connected circles. Numeric values for gene expression, protein expression and gene IDs can be found in Supplementary Tables S1, S2, S6.

To determine which functional categories of genes were affected by treatment or genotype, we performed GO analysis for each of the pairwise comparisons shown in Figure 1A and Table 1. GO analysis of both the DEPs and DETs from WT to *phyA* comparisons showed enrichment of biological processes involved in primary metabolism, specifically the GO categories “glucose metabolic process” (GO:0006006), “pyruvate metabolic process” (GO:0006090), and the “tricarboxylic acid (TCA) cycle” (GO:0006090). In contrast, GO analysis of the DEPs and DETs from comparisons between seedlings in the dark to R did not show enrichment of any of the same categories (Table 1 and Supplementary Table S5). Because of the shared enrichment of primary metabolic functions in DEPs and DETs, we looked at expression of all DE transcripts and proteins corresponding to gene IDs with annotations in glycolysis, gluconeogenesis, the TCA cycle, the glyoxylate cycle, and β-oxidation (Figure 2 and Supplementary Table S6). Of the 178 tomato genes that we identified as annotated as enzymes involved in these pathways, 48% (85) were DETs (Figure 2 and Supplementary Table S6). The overwhelming majority of the DETs involved in breaking down sugars and lipids showed higher expression in *phyA* mutants than WT regardless of light exposure. In fact, 75% (64/85) of transcripts in Figure 2 (excluding seed storage proteins) showed higher expression in the mutant than in WT. Surprisingly, the DEPs involved in the same processes, many of which are encoded by the same transcripts that were upregulated in *phyA* mutants, in fact showed lower abundance in *phyA* mutants than in WT regardless of light exposure (Figure 2). For example, eight transcripts encoding pyruvate dehydrogenase, the enzyme that converts pyruvate to acetyl-CoA and connects glycolysis to the TCA cycle, were coordinately upregulated in *phyA* mutants relative to WT but were relatively unaffected by R. For gene ID Solyc06g072580 (one of these eight transcripts annotated as belonging to the pyruvate dehydrogenase complex), the protein was also DE, but it was significantly downregulated in the mutant and again relatively unaffected by R. The consistent differential expression between WT and *phyA* mutants but not between dark and R (Figure 2) suggests both that phyA regulates primary metabolism, and also that this regulation is largely light independent in seedling development. The pattern is the strongest in the TCA cycle and β-oxidation pathway, in which most enzymes only catalyze the reaction toward breakdown of molecules into energy, whereas in glycolysis, many of the same enzymes also participate in gluconeogenesis. Additionally notable was the consistently opposite regulation between proteins and transcripts in all of these pathways. Together, these data suggest a role for phyA in the breakdown of sugars and lipids independent of light condition that is not synchronized at the transcriptional and translational levels.

**Table 1:** Biological process GO categories enriched in both DETs and DEPs across four comparisons with total significant DETs and DEPs found in each comparison.

		Differentially expressed transcripts				Differentially expressed proteins			
		
Comparison: WT-D vs. WT-R		Total DETs in comparison:			482	Total DEPs in comparison:			19
No overlapping GO categories									

**Comparison: *phyA*-D vs. *phyA*-R**		Total DETs in comparison:			1339	Total DEPs in comparison:			37

No overlapping GO categories									

**Comparison: WT-D vs. *phyA*-D**		**Total DETs in comparison:**			**5651**	**Total DEPs in comparison:**			**39**

**GO ID**	**GO description**	**A**	**S**	**E**	***p*-value**	**A**	**S**	**E**	***p*-value**

GO:0006108	Malate metabolic process	21	10	4.7	0.00935	21	2	0.04	0.00073
GO:0006099	Tricarboxylic acid cycle	36	14	8.06	0.01875	36	2	0.07	0.00215
GO:0006006	Glucose metabolic process	38	18	8.5	0.0018	38	2	0.07	0.0024

**Comparison: WT-R vs. *phyA*-R**		**Total DETs in comparison:**			**5423**	**Total DEPs in comparison:**			**62**

**GO ID**	**GO description**	**A**	**S**	**E**	***p*-value**	**A**	**S**	**E**	***p*-value**

GO:0042026	Protein refolding	10	8	2.12	0.00012	10	2	0.03	0.00046
GO:0006090	Pyruvate metabolic process	58	21	12.31	0.00621	58	3	0.19	0.00088
GO:0006006	Glucose metabolic process	38	14	8.07	0.01344	38	2	0.12	0.0068


*A is for “Annotated” – total genes annotated in tomato genome with that GO ID. S is for “Significant” – total genes in the gene set of interest (DETs or DEPs from that comparison). E is for “Expected” – the expected number of genes in the gene set of interest if no enrichment. *P*-values were found with Fisher’s Exact Test using the weighted model in topGO. No multiple test correction was applied.*

GO analysis of DEPs and DETs from WT to *phyA* comparisons also showed enrichment of molecular functions related to protein translation, including “translation elongation factor activity” (GO:0003746), “GTPase activity” (GO:0003924), and “GTP binding” (GO:0005525) (Table 2 and Supplementary Table S5). Like the metabolism related biological processes discussed above, these molecular functions were enriched in the DEPs and DETs found when comparing *phyA* mutants to WT in both the dark and R, but not in dark to R comparisons (Table 2). Like the metabolic transcripts, the majority of the translation-related transcripts identified by GO analysis were more highly expressed in *phyA* mutants while the proteins were more highly expressed in the WT (Supplementary Figure S3). Two genes, Solyc03g112150 and Solyc09g073000, both encoding organelle-specific translation elongation factors Tu, were both DE at the transcript and protein level. Both the transcript and the protein for Solyc03g112150 were more highly expressed in WT than in the mutant, an atypical pattern for these transcripts. The transcript and protein expression patterns for Solyc09g073000, however, followed the pattern established for the metabolic DEPs and DETs: the transcript was more highly expressed in the mutants and the protein was more highly expressed in WT (Supplementary Figure S3). This DE of transcripts and proteins involved in the translation machinery, specifically lower protein levels in *phyA* mutants, could help explain the lack of synchronization between transcript and protein expression, especially for cellular respiration related genes translated in the mitochondria.

**Table 2:** Molecular function GO categories enriched in both DETs and DEPs across four comparisons with total significant DETs and DEPs found in each comparison.

		Differentially expressed transcripts				Differentially expressed proteins			
					
Comparison: WT-D vs. WT-R		Total DETs in comparison:			482	Total DEPs in comparison:			19
No overlapping GO categories									

Comparison: *phyA*-D vs. *phyA*-R		Total DETs in comparison:			1339	Total DEPs in comparison:			37

No overlapping GO categories									

**Comparison: WT-D vs. *phyA*-D**		**Total DETs in comparison:**			**5651**	**Total DEPs in comparison:**			**39**

**GO ID**	**GO description**	**A**	**S**	**E**	***p*-value**	**A**	**S**	**E**	***p*-value**

GO:0045735	Nutrient reservoir activity	56	18	11.78	0.00935	56	4	0.13	7.80E-06
GO:0003746	Translation elongation factor activity	16	11	3.37	5.30E-05	16	2	0.04	0.00061
GO:0003924	GTPase activity	95	29	19.99	0.01871	95	2	0.22	0.02034
GO:0005525	GTP binding	141	44	29.67	0.00288	141	2	0.33	0.04214

**Comparison: WT-R vs. *phyA*-R**		**Total DETs in comparison:**			**5423**	**Total DEPs in comparison:**			**62**

**GO ID**	**GO description**	**A**	**S**	**E**	***p*-value**	**A**	**S**	**E**	***p*-value**

GO:0045735	Nutrient reservoir activity	56	17	11.29	0.04582	56	5	0.22	2.50E-06
GO:0030060	L-malate dehydrogenase activity	7	6	1.41	0.00039	7	2	0.03	0.00031
GO:0051082	Unfolded protein binding	55	30	11.09	1.70E-08	55	2	0.22	0.0197


*A is for “Annotated” – total genes annotated in tomato genome with that GO ID. S is for “Significant” – total genes in the gene set of interest (DETs or DEPs from that comparison). E is for “Expected” – the expected number of genes in the gene set of interest if no enrichment. *P*-values were found with Fisher’s Exact Test using the weighted model in topGO. No multiple test correction was applied.*

### Opposite Regulation of DEPs and DETs Suggests Weak Correlation Between Transcriptome and Proteome

While we found corresponding GO categories between DEPs and DETs, we found very little else coordinately regulated in the same direction (up versus down) between the transcript and protein data. First, we compared our proteomic and transcriptomic analyses to find gene IDs that were identified as DEPs and DETs in the same comparison. Of the 52 gene IDs that are common between the DEPs and DETs across the four comparisons, none were from the set comparing WT dark versus R-treated plants, and only two were from the *phyA* mutant dark versus R-treated set. These values are consistent with what we would expect from random chance shown by permutation testing (*p* = 1.0 and *p* = 0.70, respectively), suggesting no correlation between the proteome and transcriptome in the dark to R transition after 60 min of light. However, of 39 DEPs and 5651 DETs in the dark grown WT to *phyA* comparison, there were 23 overlapping gene IDs between the data sets, more than would be expected by chance (*p* = 0.0001). Similarly, in the WT to *phyA* in R comparison, of 62 DEPs and 5423 DETs, there were 29 overlapping gene IDs, again more than would be expected by chance (*p* = 0.01). Of these 21 and 29 genes, 15 genes were DEPs and DETs found in both the dark and R WT to *phyA* comparisons.

Despite more DEPs and DETs encoded by the same gene than one would expect by chance in the WT to *phyA* mutant comparisons, the overlapping DEPs and DETs did not behave similarly overall. Of the 20 gene IDs unique to one comparison, only nine were regulated in the same direction, for example higher expression in the mutant than WT, in the protein and transcript data while the remaining eleven were regulated oppositely to each other. Of the 15 overlapping gene pairs, 11 were oppositely regulated in *both* WT to mutant comparisons. These genes include many of the primary metabolism genes from Figure 2, namely glyceraldehyde 3-phosphate dehydrogenase, enolase, malate dehydrogenase, and triosephosphate isomerase and one translation elongation factor Tu (Supplementary Figure S3). Two of the four genes that show synchronized regulation between proteins and transcripts in the WT to mutant comparisons are annotated as seed storage proteins, also on Figure 2. Together, these data suggest that there is no correlation between transcripts and proteins in the dark to R transition after 60 min of light, perhaps because translation has not yet caught up with transcription. The data also suggest that while there is significant overlap between DEPs and DETs between WT and *phyA* mutants, the regulation of the transcriptome and that of the proteome are not synchronized.

It is important to note that for our protein analysis, we used 2D gel electrophoresis with which we were only able to analyze 218 protein spots, considerably fewer data points than for the transcript analysis. This limitation made the differences between our proteomics and transcriptomic results more challenging to interpret. The fact that enzymes in metabolism pathways are commonly regulated post-translationally and allosterically by the presence of certain metabolites, but are also controlled at the transcriptional level in response to environmental cues (Gaudinier et al., 2015) adds an extra level of complexity to the interpretation of the discrepancy between transcript and protein levels. In WT seedlings with normal levels of metabolic enzymes and metabolites, transcriptional upregulation may not be induced. However, in *phyA* mutants transcriptional upregulation may be induced, leading to the unsynchronized levels of proteins and transcripts observed here. Interestingly, *phyA* mutants have low expression of many translation-related transcripts (Supplementary Figure S3), suggesting that the translation process of the newly upregulated metabolism transcripts could be inhibited and contribute to explaining why we saw a lack of synchronization between transcript and protein levels. However, to fully characterize this phenomenon, more proteomic data would be needed.

### ATP Levels Are Not Affected by Differences in Carbon Flux Patterns

Given the differences in the expression of genes involved in carbon flux through the primary metabolic pathways involved in cellular energy production, we asked if ATP levels were different between the mutant and WT seedlings. Seedlings were grown as they were for RNA and protein extraction, and ATP was extracted and quantified from nine biological replicate pools for each genotype and condition. Neither treatment nor genotype had a statistically significant effect on the seedlings’ ATP content (ANOVA, *F* value = 1.185, *p*-value = 0.337), suggesting that steady state ATP levels are not affected by the differences in transcript or protein expression of the primary metabolic enzymes (Supplementary Figure S4).

### Loss of phyA Results in Abnormal Storage Protein Accumulation in Developing Seedlings

Striking in the overlap between DEPs and DETs were seed storage proteins, particularly vicilins and legumins, which are typically expressed and translated in the developing seed, then stored until germination to sustain the seedling during growth before light exposure when the plant can begin photosynthesis (Tan-Wilson and Wilson, 2012). GO category enrichment analysis identified nutrient reservoir activity as a molecular function enriched in both DEPs and DETs, mostly due to the DE of vicilins, legumins, and other seed storage proteins (Table 2 and Supplementary Table S5). Unlike DEPs and DETs involved in primary metabolism, most seed storage transcripts and proteins were regulated in the same direction; both were generally more highly expressed in the *phyA* mutant than in WT (Figure 2). Like the DEPs and DETs involved in primary metabolism and translation, this regulation was most often independent of light. The presence of more seed storage proteins in the *phyA* mutant could be indicative of slower breakdown of these proteins during germination and seedling growth, or, as the transcript data would suggest, new transcription and translation of these genes and proteins during germination and seedling growth. We saw DE of multiple proteolytic forms of several seed storage proteins, suggesting they are in different stages of catabolism in WT and mutant (Supplementary Table S1). One class of seed storage proteins, the germins, did not fit the pattern of expression. Although germins are evolutionarily related to seed storage proteins, they play diverse roles in plants unrelated to nutrient storage (Barman and Banerjee, 2015). Together, these data further support the notion that phyA regulates primary metabolism and, in addition, suggest its role in the choice of the energy storage form used in developing seedlings, largely independently of light exposure.

### Co-expression Network Analysis Supports the Role of phyA in Primary Metabolism in the Dark

**FIGURE 3:**
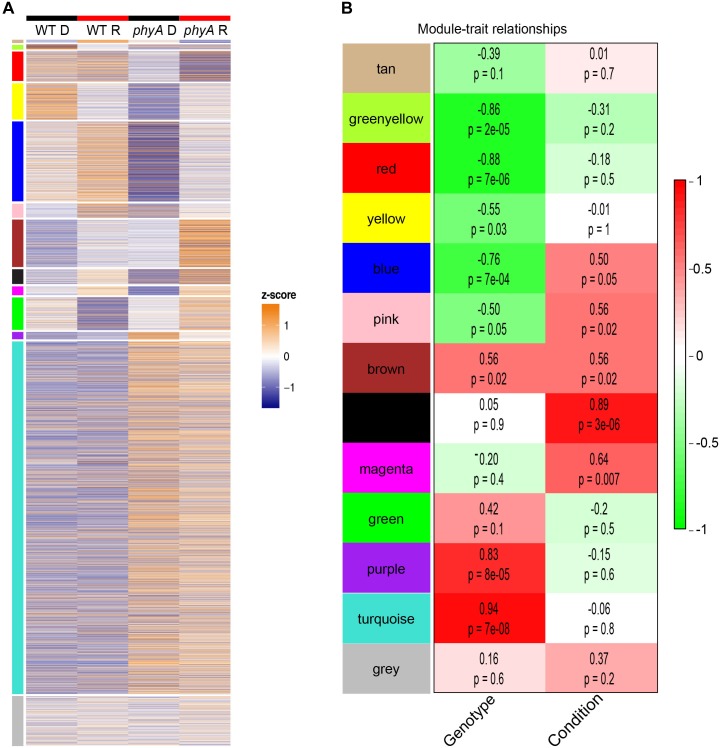
Co-expression modules show expression patterns that correlate with genotype and treatment. Modules are indicated by color (same order in **A,B)**. In **(A)** *z*-score normalized expression on a color scale of normalized read counts is shown where white represents average expression across genotype/conditions. The gray “non-module” at the bottom consists of genes that were not significantly co-expressed with other genes. In **(B)** the average expression pattern of each module (eigengene) was correlated to genotype (“genotype,” WT = 0, phyA = 1) and light treatment (“condition,” dark = 0, red = 1). The *R*^2^ value from Pearson’s correlation are indicated above *p*-values in the boxes as well as by a red to green color scale.

**FIGURE 4:**
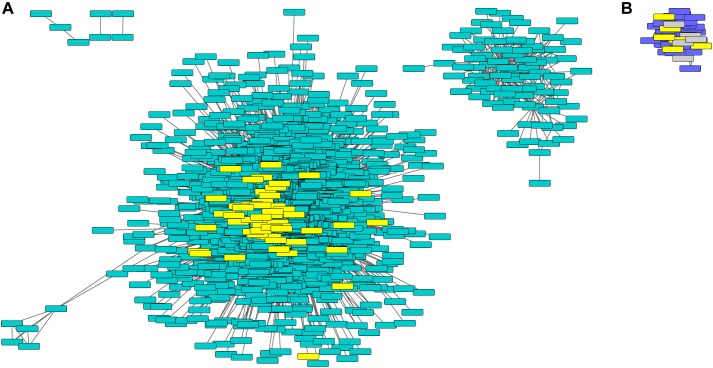
Turquoise and purple gene networks show upregulation in *phyA* mutants and contain metabolism and seed storage genes. The turquoise **(A)** and purple **(B)** gene networks in which metabolism and seed storage genes from Figure 2 were highlighted in yellow. Oleosins were highlighted in gray. Gene names and IDs, network membership, and highlighted genes can be found in Supplementary Table S7.

To gain further insight into gene-gene interactions in which phyA plays a role during seedling development, we performed co-expression analysis on the top 8000 most variably expressed transcripts in the RNA-seq data using WGCNA (Langfelder and Horvath, 2008) and found 12 co-expressed “gene modules” (Figure 3A and Supplementary Table S7). Genes in the same module are regulated similarly across genotypes and conditions (co-expressed), suggesting they may play roles in the same or related pathways. The remainder of the genes that did not cluster into co-expression modules were grouped into the gray “non-module.” Ten of the 12 modules had expression patterns that significantly correlated with genotype, condition, or both (Figure 3B). Two modules, tan and green, did not have a significant correlation with light or genotype but still had interesting expression patterns because the genes in these modules were oppositely regulated in response to light in WT and *phyA* mutants (Figure 3). From these modules, we took only those genes that were most coordinately regulated using topological overlap (TO) (≥0.2) and visualized gene network maps (Figure 4 and Supplementary Figures S5–S7) using Cytoscape (Supplementary Material) (Shannon, 2003). Of the 12 modules, nine had 10 or more highly connected genes that passed our connectivity threshold (excluding pink, black, magenta and the gray non-module). We subsequently performed GO analysis on the genes in the highly connected co-expression networks (purple, turquoise, greenyellow, red, yellow, blue, brown, tan, and green) (Supplementary Table S8).

Purple and turquoise modules contained genes with expression highly positively correlated to genotype (higher expression in mutants) suggesting negative regulation by phyA, but not significantly correlated to condition, suggesting little to no regulation by R (Figure 3). Purple and turquoise modules were distinct because exposure to R did seem to have some effect on genes in the purple module in *phyA* mutants, while light seemed to have no effect on gene expression in the turquoise module. The turquoise and purple module expression patterns matched the general pattern we also identified with the primary metabolism DETs: transcripts were more highly expressed in *phyA* than WT, but generally not different between light treatments. In fact, 43% of all tomato genes annotated as encoding enzymes in primary metabolism and 61% of the DETs encoding these enzymes (Figure 2) fell into the turquoise module (Figure 3 and Supplementary Tables S6, S7). These genes are centrally clustered in the turquoise network and have very high module membership (*p* < 0.0001, permutation test), meaning they are among the most similarly expressed genes and strongly contribute to the expression pattern that defines the network (Figure 4A). When we performed GO analysis on the genes in the turquoise network, we indeed saw that they were enriched in central carbon metabolism processes (Supplementary Table S8), such as “pyruvate metabolic process” (GO 0006090), “glucose metabolic process” (GO 0006006), and “one-carbon metabolic process” (GO 0006730). Also agreeing with our DE findings, the turquoise network was enriched in translation and translation-related processes (Supplementary Figure S3 and Supplementary Table S8), which again could help to explain the seemingly uncoordinated regulation between protein and transcript levels for the metabolic genes and others. The purple network genes were enriched in the biological processes of “sexual reproduction” (GO 0019953) and “lipid storage” (GO 0019915) by oleosin transcripts (Figure 4B and Supplementary Tables S7, S8), which encode proteins that serve as structural components of plant oil bodies where lipids are stored as energy (Schmidt and Herman, 2008). The purple network genes were also enriched in the molecular function “nutrient reservoir activity” (GO 0045735) by four vicilins, one legumin 11S globulin^1^, and one seed storage protein from the “other” category (Figure 4B and Supplementary Tables S7, S8), thus supporting the findings from DE analysis in Figure 2. In summary, co-expression network analysis, a parallel to but distinct approach from DE analysis, also identified a shift in primary metabolism (turquoise network) and energy storage (purple network) regardless of light exposure as a major effect of the *phyA* mutation.

Other modules also showed interesting expression patterns implicating phyA’s role in metabolism and energy use and storage in the dark. The greenyellow module genes had high expression in WT in the dark that decreased upon exposure to R, but expression in the *phyA* mutant was low in both conditions (Figure 3). Therefore, phyA may be necessary for expression of these genes in the dark and repression upon R. The greenyellow network was enriched in the GO category “lignin catabolic process” (GO 0046274) by laccases (lignin modifying enzymes) (Supplementary Figure S5A and Supplementary Tables S7, S8), another indicator of phyA’s role in metabolic processes in the dark. The red module showed high expression in WT regardless of light exposure, but lower expression in *phyA* mutants and a further decrease in expression upon exposure to R (Figure 3). These genes are likely positively regulated by phyA against competing signals such as other phytochromes. The red network was enriched in “carbohydrate transmembrane transport” (GO 0034219) by four *SWEET* genes, a family of genes that is involved in transporting various hexoses, including sucrose, across membranes and that plays important roles in plant growth and developmental processes (Chen et al., 2012, 2015; Feng et al., 2015) (Supplementary Figure S5B and Supplementary Tables S7, S8). Inhibited carbohydrate transport within the plant in *phyA* mutants could alter central metabolic function, seed provisioning, and growth. The yellow module genes were oppositely regulated in WT and *phyA* mutants; they were repressed in R in WT whereas in *phyA* mutants they were induced in R (Figure 3). The levels in the dark were strikingly different between mutant and WT, again highlighting the regulatory role of phyA in the dark. The yellow network was enriched in photosynthesis related biological processes, such as photosynthesis, light harvesting (GO 0009765), and photosynthesis (GO 0015979) by genes encoding chlorophyll *a* and *b* binding proteins (Supplementary Figure S5C and Supplementary Tables S7, S8). This suggests phyA plays a role not only in respiration, but also photosynthetic metabolism even before the plant is exposed to light.

The remaining modules further helped to elucidate the role of phyA in R. The tan and green networks (Supplementary Figure S6) represented complete mis-regulation of gene expression in the absence of phyA in the light (Figure 3). Both of these modules contained genes with similar expression in the dark between WT and the *phyA* mutant, as canonical phytochrome models would predict. In the tan module, genes were induced in WT by R but repressed by R in the *phyA* mutants. In the green module the opposite was true, genes were repressed by R in WT but induced by R in the *phyA* mutants. The tan network only consisted of 24 genes and had no significant GO categories, but cytochrome P450 and ATP synthases fell in this network, genes we would expect to be induced by light (Supplementary Table S7). The green network was enriched in “translation” (GO 0006412), “protein folding” (GO 0006457), and “plastid organization” (GO 0009657) (Supplementary Table S8). The green and tan co-expression modules, like those discussed above, suggest a potential for mis-regulation in the mutant of general metabolism and translation, but unlike those discussed above, the differential regulation is only seen in R.

The blue and brown modules, both of which showed misregulation of genes in response to R in WT versus *phyA* mutants, likely represent genes regulated by phyA and other members of the phytochrome family. These modules were enriched for regulatory functions such as “transcription, DNA-templated” (GO 0006351) and histone acetylation (GO 0016573) respectively and contained genes known to be involved in the phyA response such as *HY5* (Jang et al., 2013) (Supplementary Figure S7 and Supplementary Tables S7, S8).

### Mutation in PHYA Leads to Increased Seedling Growth in the Dark

Data from our genome profiling experiments (Figure 1–4) suggested to us that the altered regulation of metabolism in dark-grown seedlings should have an effect on growth. Previous studies of *phyA* mutants in Arabidopsis (Whitelam et al., 1993) and tomato (van Tuinen et al., 1995) have been somewhat ambiguous about the role phyA plays on hypocotyl elongation in the dark. Both studies show quantitative data that may suggest that loss of phyA leads to increased hypocotyl growth in the dark, but in neither of these studies was this observation the focus of rigorous analysis and discussion, nor did these authors ascribe any specific importance to this part of their results.

**FIGURE 5:**
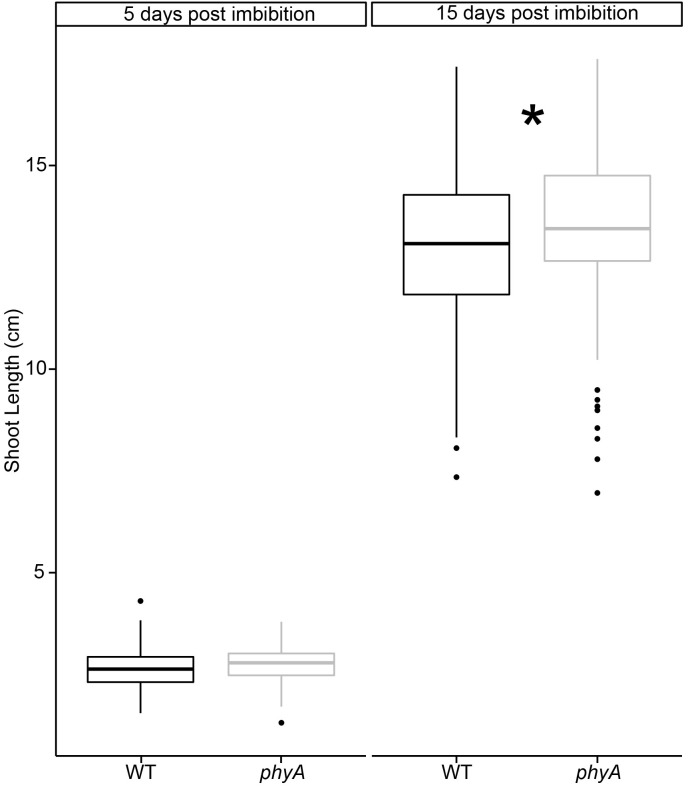
PhyA reduces shoot elongation in dark grown seedlings. Seedlings of WT and *phyA* mutants were germinated in light-excluding boxes for 2 days and checked for germination. Only synchronized seedlings with roughly 2 mm long protruding radicles were used for subsequent experiments. A subset of seedlings was removed on days 5 and 15, scanned, and their shoot length measured using ImageJ. To determine if light exposure during seedling sterilization and imbibition prior to sowing the seeds in the boxes made a difference, seed batches were either sterilized, and sown in green safe light (“total dark”) or ambient lab light (“ambient”). Subsequently, both batches were grown in complete darkness without any light treatment for germination. Two-way ANOVAs were conducted using R for day 5 and day 15. F/p-values can be found in Supplementary Table S10. Since no difference was found between ambient light and dark sterilized seeds, the data for both conditions were combined. Condition-specific data can be seen in Supplementary Figure S8. *T*-tests were performed using R to determine significance (^∗^ indicates difference between WT and mutant, *p* < 0.05, Supplementary Table S10). Sample size *N* = 552 (10–12 per genotype, imbibition condition, and time past imbibition). Regardless of a short ambient light exposure during seed sterilization, *phyA* mutants were statistically significantly taller by the end of the experiment.

In light of the findings of our genomic analyses, we decided to verify the observations from these previously published studies (Whitelam et al., 1993; van Tuinen et al., 1995). We grew tomato seedlings in controlled environmental conditions in the dark. Compared to WT, seedlings mutant in *PHYA* were initially statistically the same height as WT plants (day 5), but had statistically significantly surpassed them after 15 days of growth in the dark, according to a *t*-test (Figure 5, Supplementary Figure S8, and Supplementary Tables S9, S10). To be certain that these differences were not due to either (a) differences in germination times between mutant and WT, or (b) ambient light exposure of the seeds, during handling and sterilization procedures before planting the seeds we performed several control experiments.

To exclude the possibility that seedling lengths were influenced by germination time rather than genetics, we synchronized germination, selected seeds of comparable germination state (see Materials and Methods for details), and only used synchronized seeds for the phenotypic experiments described in Figure 5. To exclude the formal possibility that our results reflected seedlings responding to the ambient light stimulus experienced during seed sterilization in the lab prior to germination, we performed a set of control experiments in which seeds were sterilized in the dark under green safe light and compared those to seeds sterilized in ambient laboratory light conditions. After sterilization in ambient lab light or safe green light both sets of seeds were germinated and grown in the dark. We found that the difference between ambient light and dark conditions during sterilization and first imbibition had no statistically significant effect on subsequent hypocotyl growth in the dark at either day 5 or 15, as determined by a two-way ANOVA (Supplementary Figure S8 and Supplementary Table S10). Given these results, we combined the data for both analyses in Figure 5 (but show the separate results in Supplementary Figure S8).

Besides the safe green light during collection, ambient light exposure during sterilization was the only light to which these seeds were exposed as there is no light treatment required to induce uniform germination in tomato. To additionally ascertain that the use of safe green light did not inadvertently trigger photomorphogenesis, we also performed a biological assay in which we measured chlorophyll concentrations in cotyledons of seedlings grown in the various conditions and found the same very low levels of chlorophyll in all conditions (about two orders of magnitude less than light-grown tomato leaves) (Jianfeng et al., 2015) and no difference between genotypes, suggesting that the seedlings were indeed blind to the green safe light (Supplementary Table S11). Furthermore, the dark-grown seedlings across all experiments showed no obvious signs of de-etiolation.

In summary, the results suggest that phyA plays a subtle role in the dark hypocotyl growth of these seedlings at 15 days after imbibition that cannot be explained by ambient lab light exposure during sterilization or green light exposure during collection.

### PHYA-Mediated Regulation of Sucrose Transporters Affects the Seedling Phenotype

Network analysis suggested not only primary metabolism as differentially regulated in WT versus *phyA* plants, but also implicated sucrose transport as a potential mechanism by which phyA regulates early seedling development. Chen and co-workers showed that AtSWEET11, AtSWEET12, and AtSWEET15, all sucrose transporters, are involved in provisioning seeds with sugar during fruit development (Chen et al., 2015). Mutation in these genes leads to reduction in root growth in Arabidopsis seedlings that can be restored by exogenous sucrose supplementation in the growth medium (Chen et al., 2012, 2015). Since network analysis implicated sucrose transport to be significantly affected by the mutation in PHYA (red network), and since orthologs to both AtSWEET11 and AtSWEET12 were down-regulated in the *phyA* mutant (Supplementary Figure S5 and Supplementary Table S7), we hypothesized that tomato *phyA* mutants may have less well provisioned seeds, shorter roots, and a different response to supplemental sucrose than WT seedlings.

**FIGURE 6:**
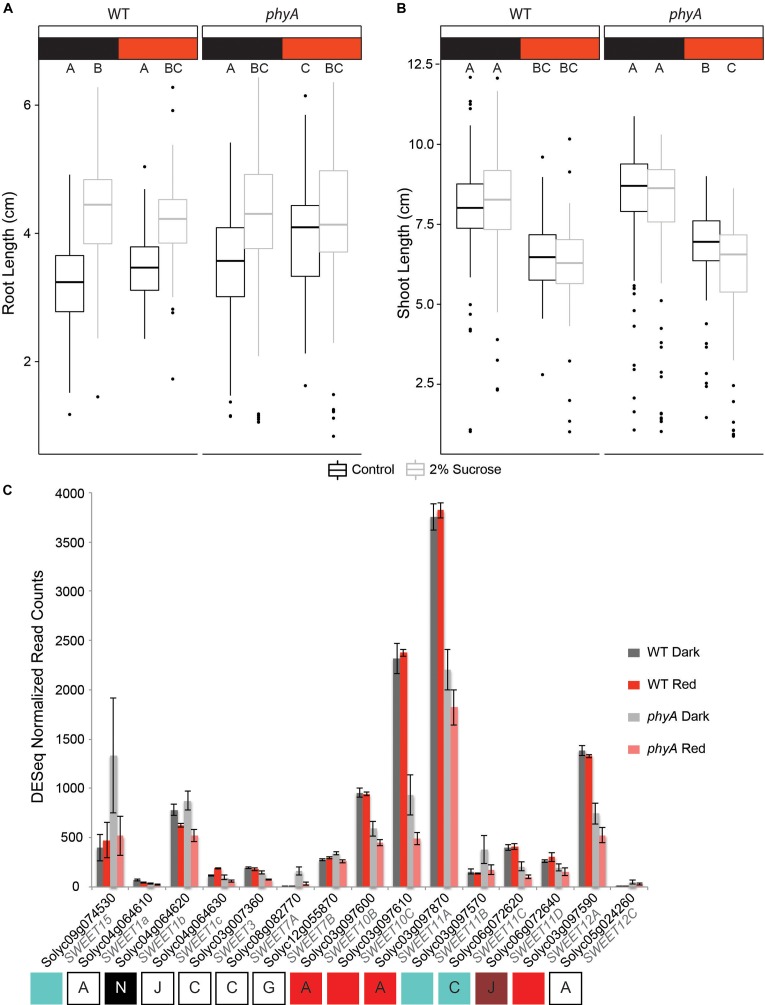
PhyA attenuates root growth in R and interacts with sucrose response. Root length **(A)** and shoot length **(B)** of germination-matched 5 day old seedlings was measured using Image J software. Three-way (factorial) ANOVAs were conducted using R. *F/p*-values can be found in Supplementary Table S13. Tukey *post hoc* analysis was performed using R to assign groups (letters). Data points not connected by a common letter are statistically significantly different from each other. Sample size *N* = 765 (94–96 per genotype, light environment, and sucrose treatment). Box plots in A and B indicate range of data, including the median (bold line), 25th and 75th percentiles (lower and upper box limits), 5th and 95th percentiles (lower and upper whiskers, respectively), and outliers (indicated with dots). **(C)** Gene expression values were extracted from the RNA-seq analysis using normalized read counts from DESeq analysis. Letters and colored boxes underneath the graph are connected with each gene and indicate the sector in which the gene was located on the Venn diagram analysis of DE genes (Figure 2B) and the module (Figure 4) the gene belongs to (if any).

When we grew both WT and *phyA* mutants on MS agar medium with or without 2% sucrose, we observed a significant increase in root length on sucrose-supplemented medium in both WT and mutant seedlings in darkness (*p* < 2e-16). *phyA* mutants grown in R did not show a statistically significant increase in root length in response to sucrose, while WT in R did (Figure 6A and Supplementary Tables S12, S13). Interestingly, and contrary to our hypothesis, the non-responsiveness of *phyA* to sucrose supplementation in R was caused by increased root growth of the mutant in R on non-supplemented MS medium, not by shorter roots on sucrose-supplemented medium. Since both the genotype:sucrose and sucrose:light interaction terms of a three-way ANOVA were highly significant (Supplementary Table S13), the data suggest that in R (and with no sucrose supplementation), active phyA keeps root growth at the same levels as they are in darkness, while in the absence of its activity, R increases root growth. At the same time, the data suggest that phyA works antagonistically with sucrose in root elongation in R.

We also examined the effect of the *phyA* mutation with respect to its role in the regulation of sucrose transport during hypocotyl elongation in both darkness and R (Figure 6B and Supplementary Table S12). As expected, R inhibited hypocotyl growth as compared to growth in darkness for both WT and *phyA* mutants (Supplementary Table S13). While sucrose had no effect on the WT response in both light conditions, there was a statistically significant genotype:sucrose interaction effect that reduced shoot growth in *phyA* even further than R alone (Figure 6B and Supplementary Table S13), while having no such statistically significant effect on the WT. These data suggest that active phyA plays a role in the promotion of a sucrose-dependent response in the shoot. Taken together, the experiments suggest that phyA’s strong effect on the regulation of sucrose transporters (Figure 6C) has a small, but significant effect on the seedling phenotype (Figure 6A,B).

## Discussion

The basic “rule” of phy activity states that illumination with specific wavelengths turns the inactive Pr form of phy into the active Pfr form, which is physiologically active and triggers photomorphogenesis (Rockwell et al., 2006). Phy responses can be distinguished and influenced by their specific fluence requirements, their ability to use R or FR light for activation, the time it takes from light perception to response, the rate of *de novo* synthesis of Pr, stability and degradation of Pfr, and dark reversion from Pfr back to the Pr form (Sineshchekov, 1995).

Our genomic and bioinformatic approaches (Figure 1–4) to test if phyA plays a role not only during transition to light development but also during the skotomorphogenic phase of seedling growth resulted in new hypotheses that we subsequently tested phenotypically (Figure 5, 6). Intriguingly, the observation that seedlings of both Arabidopsis and tomato *phyA* mutants grow taller hypocotyls in the dark when compared to their isogenic WT lines (Figure 5 in this study, and possibly Figure 2 in Whitelam et al., 1993, and Figure 3 in van Tuinen et al., 1995) suggests that phyA also exerts a function in seedlings that have never been exposed to light. As phyA is the only phytochrome that can be activated by FR or by very low fluence, phyA is able to regulate growth with very low levels of active Pfr (Sineshchekov, 1995). Several possibilities exist by which phyA could have an effect in dark-grown seedlings: first, phyA is activated when seeds are first imbibed in ambient light and then germinated in the dark, as might happen if a dry seed is first exposed to moisture, then buried by animals, wind or erosion under soil or leaf litter, and then germinates in darkness. There is strong evidence for this type of light activation of phyB in seeds in *A. thaliana* (Mazzella et al., 2005; Leivar et al., 2008). A second possibility for the effects of active phyA in dark grown seedlings could be that phyA protein from the mother plant directly initiates a signaling cascade in the developing embryo, or that phyA is activated in the embryo itself while still on the mother plant setting in motion a signal transduction cascade whose intermediaries are preserved through dormancy and into the germinating seed. A third possibility to explain phyA functions in dark grown seedlings might be that the active Pfr form is produced in very small quantities by stochastic conformation changes of the chromophore or that inactive Pr can bind to PIFs at efficiencies several magnitudes lower than in light but still with enough strength or frequency to elicit certain phenotypic effects.

The first possibility proposed above, in which phyA is activated during seed sterilization/imbibition in the light followed by germination in the dark, would require phyA to be present in the seed during imbibition or to be produced and activated rapidly enough upon imbibition to exert its function after the seeds are sown. However, phyA, unlike phyB, is not present in Arabidopsis seeds and only starts to accumulate after at least 4 h post-imbibition regardless of light conditions (Shinomura et al., 1996; Sharrock and Clack, 2002), suggesting that phyA itself is unlikely to be activated in dark grown seeds that have only been exposed to light during a short imbibition period. Assuming phyA is also absent in tomato seeds, any light exposure during sterilization, imbibition or planting of seeds could not have resulted in phyA mediated signaling and therefore could not explain expression or phenotypic differences as reported in our study. Additionally, our data show no difference in growth or greening in the dark between tomato seedlings exposed to ambient light during sterilization and those kept entirely in the dark, refuting the possibility that activation of phyA during the experimental set-up is responsible for the observed differences (Supplementary Figure S8 and Supplementary Tables S10, S11). Our qPCR validation experiments performed with three biological replicates each of dark sterilized and ambient light sterilized seeds showed congruent responses (Supplementary Figure S2), further refuting this possibility.

If phyA is indeed absent from the mature tomato seed, then, as proposed in the second possibility above, active phyA in the developing seed or in the fruit of the parent might trigger downstream cascades that would then be active in the germinating seed and seedling. Mazzella et al. (2005) suggested that seed-activated phyB triggers a signal in the seedling that results in the suppression of *ABSCISIC ACID INSENSITIVE3* (*ABI3*) expression and reported elevated levels of ABI3 in dark-grown Arabidopsis *phyB* seedlings. These authors hypothesized that high ABI3 expression in the Arabidopsis *phyB* mutant might be responsible for putting the seedling in a quiescent stage in the absence of photomorphogenesis-inducing light conditions. Interestingly, our data (Supplementary Table S2) show that the *ABI3* homolog (Solyc06g083590) in dark-grown tomato *phyA* mutants is also highly transcriptionally upregulated (∼15-fold) compared to WT. It is possible that the absence of phyA in tomato seedlings has a similar effect on inducing quiescence as the *phyB* mutation does in Arabidopsis. Since phyA, at least in Arabidopsis, is unlikely to be the direct signaling agent between seed and seedling, a more likely scenario is one in which light-activated phyA-mediated signaling occurred during seed development and is propagated to the germinating seed and seedling through an intermediary, such as ABI3. Active phyA signaling could be initiated by phyA in the fruit of the mother plant or by phyA in the developing seed, an activity that is then lost during maturation or dormancy. Our data support this scenario of signaling from developing seed to germinating seedling, but our results cannot determine whether the active phyA signal comes from the mother plant, the seed itself, or both.

Lastly, the third possibility suggested above where either minute amounts of active Pfr are formed in the dark by stochastic conformation changes, or PIFs are activated by the normally inactive Pr form at very low rates, is also consistent with our observations.

### Carbon Flux Is Regulated by phyA

Regardless of how a phyA-dependent response cascade is transmitted to dark-grown seedlings, we asked what the consequences of the lack of phyA was on skotomorphogenic development. To further investigate the role of phyA during dark growth we profiled both the transcriptome and proteome of WT and *phyA* seedlings. Mutants grown in the dark exhibited stark gene expression differences from WT (Figure 1 and Supplementary Tables S1, S2). In fact, more differences were seen in the genotype comparison in the dark than between WT seedlings grown in the dark compared to those exposed to R, strongly supporting a role for phyA during dark growth. Our results suggest that phyA has a surprisingly wide range of functions in dark grown seedlings, and specifically highlight those that are involved in the regulation of carbon flux in central metabolism (Figure 2 and Table 1). These data suggest that the enzymes of glycolysis, β-oxidation, and the TCA cycle are transcriptionally upregulated in the mutant, presumably positioning the seedling for increased glucose and fat breakdown, and thus energy production. In adult Arabidopsis plants, phytochromes in general and particularly phyB and phyD, have been found to be important for regulating carbon flux, resource use, and biomass accumulation in photoautotrophic growth (Yang et al., 2016). This regulation could be useful to the plant to conserve resources in conditions less favorable for photosynthesis, such as growing in the shade. In dark-grown tomato seedlings – another scenario in which carbon sources are limited – phyA may play a similar role helping to adequately allocate resources before the onset of photosynthesis.

Involvement of phytochromes in light-responsive repression of genes involved in respiration has been previously proposed (Igamberdiev et al., 2014). In contrast, our data show phyA repression of respiration genes in the dark and little to no repression of respiration genes by 60 min of R. It is possible that more than 60 min R treatment would uncover phytochrome involvement in light repression of respiration genes in addition to phytochrome involvement in the dark.

If indeed transcriptional upregulation in catabolic enzymes leads to increased energy formation, we hypothesized that ATP levels might also be increased. We measured free ATP in seedlings in the dark and in R but observed no significant differences between genotypes or conditions (Supplementary Figure S4), and found that ATP levels were in the same approximate range as measured previously in leaves of tomato (Guo et al., 2016), suggesting that ATP is used at the same rate as it is produced in both genotypes. If carbon flux through primary metabolism is increased in *phyA* but not used for net energy production, we asked where this carbon might be used instead and where any additional ATP produced in the mutant might be used. We noticed that transcripts for storage proteins as well as the storage proteins themselves, including legumins and vicillins, were strongly upregulated in *phyA*. Since translation requires ATP, it is possible that any additional ATP produced in the TCA cycle is used for the translation of storage proteins. This also suggests that carbon might be redistributed from carbohydrate and fat reserves into protein reserves in the mutant, a process that phyA would restrict in the WT. Glycolysis and the TCA cycle provide the majority of carbon for amino acid synthesis. Indeed, we found at least five amino acid synthase genes to be transcriptionally upregulated in *phyA* in the dark compared to WT (Supplementary Table S2), including those for Ala (Solyc11g068540), Cys (Solyc10g012370), Trp (Solyc096190), Thr (Solyc06g062840), and Glu (Solyc12g041870), an amino acid overrepresented in storage proteins (Richter, 1988), suggesting that increased storage protein formation requires additional amino acid synthesis. The notion of altered carbon redistribution in *phyA* is further supported by the upregulation of the glyoxylate cycle in the mutant, which uses malate synthase and isocitrate lyase to sidestep the TCA cycle, thereby circumventing its NADH yielding/CO_2_ releasing steps (Kornberg and Beevers, 1957) resulting in less ATP production compared to the full oxidation of pyruvate if the TCA cycle is completed in full. Malate synthase is predominantly active during germination and pre-photosynthetic seedling establishment, aiding in the breakdown of storage fats in the dark (Trelease and Doman, 1984; Smith and Leaver, 1986) with the option to eventually convert malate to phosphoenolpyruvate, and eventually to carbohydrate (Kornberg and Beevers, 1957). In *phyA* the carbon-conserving glyoxylate pathway may be used to catabolize fats to malate and then instead of producing carbohydrates, shunt carbon into amino acid and eventually storage protein production.

An interesting additional observation may support this notion of the rebalancing of energy stores in *phyA*: In Arabidopsis *SNF1-RELATED KINASE 1* (*SnRK1*) has been shown to be a central player in the transcriptional reprogramming in response to declining energy levels leading to the induction of catabolic processes and the reduction of some anabolic reactions (Crozet et al., 2014; Pedrotti et al., 2018). The reciprocal best BLAST hit ortholog of Arabidopsis SnRK1 in tomato (Solyc02g067030) falls in the turquoise module and another SnRK1 ortholog (Solyc03g115700) is significantly upregulated in dark grown *phyA* seedlings compared to WT seedlings in the dark (Supplementary Tables S2, S7). We observed DE of many of the same genes identified as DE in an *A. thaliana snrk1* mutant, particularly in our WT to mutant comparisons (Pedrotti et al., 2018). It is, however, unclear at this point if this interaction is causative or correlative.

Taking together the observation that the mutant redistributes carbon into storage protein (Figure 2), and that free ATP levels are unaffected by the mutation (Supplementary Figure S4), it appears that phyA plays a central role in how energy is provisioned during seed and early seedling development, potentially preparing the seedling for the transition from skotomorphogenesis to photomorphogenesis. Our finding that *phyA* seedlings grown in the dark are slightly taller than WT (Figure 5) supports the idea that metabolic differences might result in phenotypic differences. Extended dark growth in a seedling may induce an energy saving, quiescent state through modulating expression of genes like *ABI3* and *SnRK1*, carefully balanced by phyA. Because interpreting low light signals is key to a plant’s ability to understand when it needs to enter an energy saving state, phyA, as a very low fluence activated, light-labile phytochrome makes intuitive sense as that signal interpreter. Inducing this type of energy saving state also depends on carbon presence and signaling, both potentially regulated by phyA through primary metabolism and sugar transport.

### The Seedling Phenotype Is Fine-Tuned by phyA-Regulated Carbohydrate Transport

Both differential expression and co-expression network analyses (Figure 6C and Supplementary Figure S5B) indicated a role for phyA in the regulation of carbohydrate-, specifically sucrose- transporters in tomato seedlings. The four SWEETs that fell in the red network and a few others had expression levels that were reduced by roughly 50% in the *phyA* mutant (Supplementary Table S2 and Figure 6C). Interestingly, in several SWEETs the mutant showed a reduction in gene expression in response to R that was not seen in the WT response (Figure 6C), suggesting that phyA is required to maintain expression levels and prevent reduced expression in response to R. It is likely that this R-mediated reduction in expression is regulated by one or more different R-responsive light receptors, such as another phytochrome. In this case, our data suggest that phyA acts antagonistically to such other photoreceptors, possibly to reach the perfect balance in expression of their target genes.

The naïve interpretation of reduced transcript levels of SWEETs leading to a reduction in sucrose transport to the sink organ would be to expect reduced root growth if sucrose delivery equaled growth promotion. However, past experiments using Arabidopsis have shown that a biliverdin reductase-induced reduction in phytochrome activity in R-grown Arabidopsis seedlings leads to a significant root length increase, and that Arabidopsis *phyA* mutants grown in blue light also have longer roots (Costigan et al., 2011). Our observations of increased root length in *phyA* mutants in R (Figure 6A) support this finding. The fact that root length is not affected by R in WT seedlings, while growth is increased in the R-grown mutant compared to dark-grown mutant seedling but only in no-sucrose control conditions, mirrors the expression patterns of some of the carbohydrate transporters in our experiment (*SWEET10B, SWEET10C, SWEET11A, SWEET11C, SWEET12A;* Figure 6C). Additionally, in Arabidopsis, *AtSWEET11* and *AtSWEET12* are positive regulators of root growth (Chen et al., 2012). The same study also showed that the effects of mutation in these genes can be restored by the exogenous supply of sucrose.

According to the observations by Costigan et al. (2011) and Chen et al. (2012) we would expect a synergistic or at least additive response in phyA mutants grown with sucrose supplementation resulting in longer roots than WT because (a) the mutation increases root length in Arabidopsis (Costigan et al., 2011) and (b) sucrose increases root length in Arabidopsis (Chen et al., 2012). By contrast, we observed an increase in root length in *phyA* in R but no additional or synergistic root length increase through 2% sucrose supplementation. Additionally, the shorter size of the mutant hypocotyls in R on sucrose supplemented medium (Figure 6B) suggests that phyA counter-acts the sucrose-dependent decrease in hypocotyl growth. Given these results, one interpretation might be that sucrose delivery has to be balanced between shoot and root in order for the seedling to achieve optimal growth and establish itself during the transition from skotomorphogenesis to photomorphogenesis. Our working model therefore is that WT phyA promotes root growth through the transcriptional upregulation of SWEETs, while also inhibiting root growth through other pathways. This antagonistic function could allow for precise balancing of root elongation depending on light and carbon availability, whereby root growth is only enhanced when enough sucrose is available, while it is shut down when sucrose is limiting. What might be the advantage of such regulation for the plant? When sucrose newly becomes available, for example via transport from the cotyledons as they are beginning to green up and starting to undergo photosynthesis after perceiving light for the first time, root growth might be expected to be the main developmental priority for the plant, since the seedling is now autotrophic and the next phase in seedling establishment could be the development of a robust root system. However, since the seedling also needs to invest energy in shoot development and primary leaf growth and may also still need to continue to expand its cotyledons, unrestricted root growth might divert too much of the photosynthate to the roots and not leave enough for the shoot, especially when sucrose is limited. Our data support a scenario where phyA helps orchestrate a network of players, including the SWEETs, in an effort to balance root and shoot growth in response to sucrose availability.

## Conclusion

Genomics coupled with transcriptional network analysis is a powerful tool to formulate new hypotheses for functional analysis, especially to uncover subtle phenotypic responses that are not easily seen by eye. Our results show that phyA is involved in a multitude of functions in tomato seedlings during the transition from skotomorphogenesis to photomorphogenesis. While it remains unclear if phyA itself is the direct source of this activity in dark-grown seedlings or if the phyA-induced signal transduction activation in the developing seed is transmitted to the seedling via a downstream signal, such as ABI3. During dark growth phyA appears to play a role in the orchestration of carbon flux and energy provisioning. Our data suggest that phyA is involved in balancing rapid etiolated elongation growth with slower, energy-conserving axis growth. This strategy may allow the plant to optimize its growth rate depending on the timing of light availability. Immediately after transition to light growth, phyA continues its role in balancing seedling growth through its involvement in sucrose transport and the partitioning of sucrose within the seedling. This could be validated in the future using knock-out or knock-down mutants of sucrose transporters, or by measuring compartmentalization of sugars within the plant. Overall our data suggest that phyA through its network of interacting proteins has properties that can both accelerate or reduce growth, depending both on the most likely future condition - for the seed to eventually reach light – and on actual conditions during prolonged etiolated growth or after reaching the light.

## Materials and Methods

### Plant Materials and Growth Conditions

*Solanum lycopersicum* seeds of cultivar MoneyMaker (Gourmet Seed, Hollister, CA, United States) and homozygous *phyA* mutant *(fri)* in the MoneyMaker background (Tomato Genome Resource Center, Davis, CA, United States), which have been described previously (van Tuinen et al., 1995), were used in this study. For RNAseq experiments, seeds were surface sterilized with 10% bleach for 15 min in ambient lab conditions and then sown on saturated, sterile filter paper in light-excluding plastic boxes inside a dark growth chamber at 25°C. Five-day old seedlings of similar height were harvested in green light, and flash-frozen in liquid nitrogen. Half of the seedlings were exposed to red light treatment (660 nm LED custom display, 10 μmol m^-2^ sec^-1^) before harvesting and flash freezing. Samples were stored at -80°C until RNA and protein extraction.

Tissue was grown in six biological replicates of which all six were used for proteomic analysis, and four of these same biological replicates were used for RNA-seq analysis.

### Protein Extraction and Rehydration

Proteins were extracted and rehydrated following a previously established protocol with slight modifications for plant tissue (Fields et al., 2012). Specifically, for each biological replicate, 300 mg of frozen seedling tissue sample was ground into a fine powder in liquid nitrogen with a mortar and pestle. Proteins were precipitated by adding 1 mL 10% trichlorocacetic acid in acetone with 0.07% β-mercaptoethanol by volume. The samples were incubated at -20°C for 2 h and then centrifuged before the pellets were washed with cold acetone with 0.07% β-mercaptoethanol by volume. This process was repeated until pellets were colorless. The pellets were then air dried and stored at -80°C. The pellets were resuspended in rehydration buffer [7 mol^-1^ urea, 2 mol -1 thiourea, 2% cholamidopropyl-dimethylammonio-propanesulfonic acid (CHAPS), 2% nonylphenoxylpolyethoxyl ethanol (NP)-40, 0.002% Bromophenol Blue, 0.5% IPG buffer and 100 mmol^-1^ dithierythritol] with vortexing and allowed to incubate at room temperature for 30 min. Samples were spun, the supernatants with rehydrated protein were collected, and the pellets were discarded. Protein concentrations were determined using a 2D Quant kit (GE Healthcare Life Sciences, Piscataway, NJ, United States) according to manufacturer instructions.

### 2D Gel Electrophoresis for Protein Separation

Protein samples were separated by 2D gel electrophoresis as previously described (Fields et al., 2012). Briefly, samples were separated by isoelectric point using IPG strips (pH 4–7, 11 cm; GE Healthcare) and an isoelectric focusing cell (Bio-Rad, Hercules, CA, United States). The strips were then incubated in equilibrium buffer [375 mmol-1 Tris-base, 6 mol-1 urea, 30% glycerol, 2% SDS and 0.002% bromophenol blue], placed on top of 11.8% polyacrylamide gels, and run at 200 volts for 55 min. Gels were stained overnight with Coomassie Blue (G-250), distained with Milli-Q water, and imaged with an Epson 1280 transparency scanner (Epson, Long Beach, CA, United States).

### Protein Quantification

Digitized images from biological replicates of each genotype/condition were fused into a composite image using Delta 2D (version 3.6; Decodon, Greifswald, Germany) and spot boundaries were identified. The relative spot volume for each spot on a gel was normalized against total spot volume in the image. To determine which proteins changed in volume due to mutation in *PHYA* or red light treatment, a Student’s *t*-test was performed on normalized spot volumes (*p* < 0.01).

### Protein Identification With Mass Spectrometry

All visible spots were excised from the gels using a gel corer. Gel plugs were distained, dehydrated, and digested with 11 ng μl^-1^ trypsin (Promega, Madison, WI, United States) overnight at 37°C. Digested proteins were eluted and combined with matrix solution, then spotted onto an AnchorchipTM target plate (Bruker Dalton’s Inc., Bilerica, MA, United States) in duplicate. One replicate of the spotted proteins was washed with 0.1% trifluoroacetic acid (TFA) and recrystallized using an acetone/ethanol/0.1%TFA (6:3:1) mixture. Peptide mass fingerprints (PMFs) were obtained using a matrix assisted laser desorption ionization tandem time-of-flight (MALDI TOF-TOF) mass spectrometer (Ultraflex II; Bruker Dalton’s Inc.). We used flexAnalysis (version3.0; Bruker Dalton’s Inc.) to detect peptide peaks (PMF threshold of 500 ppm for MS and LIFT threshold of 0.6 Da for MS/MS). Porcine trypsin (Sigma-Aldrich) was used for internal mass calibration. Proteins were identified using Mascot protein identification software (version 2.2; Matrix Sciences Inc, Boston, MA, United States) and combined PMFs and tandem mass spectra in a search against the NCBI Solanum protein database.

### RNA Extraction and Sequencing

Frozen tissue samples were ground into a fine powder in liquid nitrogen with a mortar and pestle. For each biological replicate, 100 mg of seedling tissue was pooled (∼5 seedlings) for each genotype/condition, and RNA was extracted using the RNeasy Plant Mini Kit (Qiagen) according to the manufacturer’s instructions. Library construction and sequencing was conducted by BGI Americas (Cambridge, MA, United States) using paired end 100 bp reads on an Illumina Hi-Seq 2000 instrument. All data are available for public use at NCBI’s short read archive http://www.ncbi.nlm.nih.gov/sra/SRP072067.

### RNA-seq Differential Expression Analysis

RNA-seq reads were mapped to the SL2.4 version of the tomato genome with ITAG2.4 genome annotation from Sol Genomics^2^ using both Tophat2 (Trapnell et al., 2013) and HISAT2 (Kim et al., 2015). DETs were identified by three different methods with default parameters: DESeq (Anders and Huber, 2010), edgeR (Robinson et al., 2010), and Cuffdiff2 (Trapnell et al., 2013). Raw counts were determined from bam files (sorted by name) with HTseq-count (Anders et al., 2015) for input into DESeq and edgeR, whereas bam files (sorted by position) were directly input to Cuffdiff2. Our final set of DETs were those that were identified as significantly DE (corrected *p*-value < 0.05) regardless of mapping strategy by at least two of three DE methods and for which gene identifiers (Solyc numbers) existed in the Sol Genomics Networks database.

### Co-expression Analysis With WGCNA

The normalized read counts from DESeq were log transformed [log_2_(normalized read count + 1)] and used as input for WGCNA (Zhang and Horvath, 2005) in the software package R to identify co-expressed genes. The 8000 genes with the highest normalized count variance across the 16 samples (two genotypes, two conditions, four biological replicates) that had gene identifiers were used. Beta was set to 20 for the adjacency function. Modules were obtained based on TO and eigenvectors representing expression of each module were correlated to genotype and condition (WT = 0 and phyA mutant = 1, dark = 0, 60 min R = 1). Those members of a module with TO with at least one other member of ≥ 0.2 were exported in an edge file and included in networks modeled in Cytoscape (Shannon, 2003).

### GO Analysis

Gene lists were tested for GO enrichment using topGO in R (Alexa and Rahnenfuhrer, 2010). *P*-values are from Fisher’s Exact Tests using the weighted model (Alexa et al., 2006). All categories with *p*-values < 0.05 were presented. The GO annotations for *S. lycopersicum* genes from the Panther Classification System^3^ (downloaded May 2017) were used as the “gene universe.”

### Permutation Testing

Permutation testing was done in R. When looking at overlap between DEPs and DETs in our data, a set of 23,876 possible DETs from HISAT2 mapping with a subset of 218 DEPs from total spots on the 2D gels were used. For example, in the WT in dark versus WT in R comparison, we identified 27 DEPs and 482 DETs. For this permutation test, 27 DEPs were chosen at random from the subset of 218 and 482 DETs were chosen at random from the set of 23,867 and the overlap of genes between the two randomly chosen groups was calculated. This was repeated 10,000 times and *p*-values were calculated by percentage of permutations at or greater than the observed overlap. This permutation testing was completed for each DE comparison randomly drawing the appropriate number of DEPs and DETs. The same approach was used to compare overlap between DETs found in *A. thaliana* (Tepperman et al., 2006) and in *S. lycopersicum* in our experiment under very similar conditions. For the set of possible DETs from *S. lycopersicum*, the set of 23,876 from HISAT2 mapping was again used. For the set of possible DETs from *A. thaliana*, a subset of 12,206 possible DETs were used which corresponded to the *A. thaliana* genes on the microarray (Tepperman et al., 2006) that have reciprocal best blast hits with *S. lycopersicum* annotated genes. Again, 10,000 permutations were used to calculate a *p*-value.

### Seedling Length Measurements During Dark Growth

In six separate biological replicate experiments (*N* = 10–12 per genotype, per time point, precondition, per biological replicate, total *N* = 552), *phyA* mutants and MoneyMaker seedlings were sterilized in 50% bleach for 15 min rinsed, and placed on saturated filter paper in light-excluding plastic boxes in a dark incubator at 25°C. Sterilization and planting either happened in ambient lab light or in darkness with green safe light supplementation. Two days after sowing, seeds were checked for germination under green safe light. Only those seeds with slightly protruding radicles (∼ 2 mm long) were considered germinated and transferred to a new box for the experiment. Seedlings were grown in the dark for 15 days, with a subset of them collected at day 5 and day 15 under green safe light, and scanned on a flat-bed scanner. Root and shoot lengths were measured with ImageJ.

### De-Etiolation Bioassay

Chlorophyll was extracted from cotyledons of 16 days old seedlings grown in the same conditions as previously described. Chlorophyll extraction was done overnight in methanol and measured with a spectrophotometer. Chlorophyll was quantified according to published procedures (Porra et al., 1989).

### Sucrose Response

*phyA* mutants and MoneyMaker seedlings were sterilized in 50% bleach for 15 min, rinsed, and placed on saturated filter paper in light-excluding plastic boxes all while working under green light. Boxes were then placed in a dark incubator at 25°C. After ∼48 h, germinated seeds with radicles extending approximately 2 – 5 mm from the seed were transferred under green light to 0.5X MS medium (0.5% agar) with or without 2% sucrose. At 5 days old, half of the seedlings were exposed to continuous R (660 nm LED custom display, 10 μmol m^-2^ sec^-1^) for 24 h. Seedlings were collected and scanned at 6 days old. Roots and shoots were measured using ImageJ.

### ATP Quantification

*phyA* mutants and MoneyMaker seedlings were sterilized in 50% bleach for 15 min, rinsed, and placed on saturated filter paper in light-excluding plastic boxes all while working under green light. Boxes were then placed in a dark incubator at 25°C. After 5 days, dark grown seedlings were collected in pools of 5, weighed, and flash frozen under green light. The remaining seedlings were exposed to 1 h of red light (660 nm LED custom display,10 μmol m^-2^ sec^-1^) before collection, weighing, and flash freezing. Frozen tissue was ground in a mortar with a pestle and liquid N. Samples were boiled for 10 min in 2 mL of Tris-HCL Buffer (50 mM, pH 7.8). ATP standards were prepared in the same buffer (5X dilution series from 100 μM to 6.4 nM) with Tris-HCl used as blank. After boiling, samples were spun at 4°C for 10 min at 14,000 rpm in a table top centrifuge. Samples and ATP standard were loaded into a plate provided with the ATPlite 1step kit (PerkinElmer, Waltham, MA, United States), substrate solution was added according to kit manufacturer’s instructions, and luminescence was measured by a SpectraMax M2 microplate/cuvette reader (Molecular Devices, Sunnyvale, CA, United States).

### QPCR Expression Validation

Tomato seeds were surface sterilized with 50% bleach for 15 min in ambient lab light or in darkness (green safe light), rinsed, and then sown on saturated, sterile filter paper in light-excluding plastic boxes inside a dark growth chamber at 25°C. Five-day old seedlings of similar height were harvested in green safe light, and flash-frozen in liquid nitrogen. Half of the seedlings were exposed to R treatment for 60 min (660 nm LED custom display, 10 μmol m^-2^ sec^-1^) before harvesting and flash freezing. Total RNA from dark grown and 60 min R grown tomato seedlings was extracted (3 μg for ambient light sterilized and 1 μg for dark sterilized) using a QIAGEN RNeasy kit as per the manufacturer’s instructions. Reverse transcription was performed using an iScript cDNA Synthesis kit (Bio-Rad) and a thermocycler program of 25°C for 5 min, 46°C for 20 min and 95°C for 1 min. QPCR was performed on a Bio-Rad Mastercycler C1000. The reactions were carried out using iTAQ Universal SYBR Green Supermix (Bio-Rad) and incubated at 95°C for 3 min, 40 cycles of 95°C for 10 s, and 60°C for 30 s followed by 95°C for 10 s. *SAND* (Solyc03g115810) and *RPL2* (Solyc10g006580) genes were used for normalization. PCR specificity was checked by melting curve analysis, and data were analyzed using the 2^-ΔΔCt^ method (Livak and Schmittgen, 2001). Statistical analysis was performed using *t*-tests on log10 normalized expression values. All primers for this RT-qPCR are specified in Supplementary Table S14.

## Significance Statement

Transcriptomic, proteomic, and co-expression network analysis suggest that phytochrome A plays a role in primary metabolism in dark grown seedlings. Physiological experiments further show that phyA, via the SWEET family of sucrose transporters, fine-tunes root and shoot elongation in the transition from dark to light growth.

## Author Contributions

AM conceived the original research experiments. KC, SB, DA, and AM performed the experiments and analyzed the data. LT supervised the proteomics experiments. KC and AM wrote the article with contributions of all the authors.

## Conflict of Interest Statement

The authors declare that the research was conducted in the absence of any commercial or financial relationships that could be construed as a potential conflict of interest.

## Footnotes

1 Solyc09g025210 is mis-annotated as ADH2 on the locus page on solgenomics.org, but the gene feature details on solgenomics.org and a protein BLAST search using the MASCOT search engine identify the gene as Legumin11Sglobulin. The Panther database assigns the “nutrient reservoir activity” GO category to this gene.

2 https://solgenomics.net/

3 www.pantherdb.org

## Supplementary Material

Figure S1 Principal components analysis of RNAseq data shows clustering of biological replicates. RNAseq data from DESeq analysis of four biological replicates of each genotype and condition combination was analyzed by principal components analysis. *X*-axis shows principal component 1 (PC1) and *Y*-axis shows principal component 2 (PC2). Dots represent individual biological replicates from the sample groups WT in dark (blue), WT in R (purple), *phyA* in dark (pink), *phyA* in R (green).

Figure S2 QPCR validation of expression patterns of five genes from RNAseq analysis. Normalized gene expression of five genes from two qPCR experiments (first three columns) and RNAseq experiment (last column) is shown. QPCR experiments were performed to replicate RNAseq experiment, one with seed sterilization in ambient lab light like done for RNAseq (ambient) and the other with seed sterilization in dark with green safe light (total dark) as a control. QPCR expression was calculated with the 2^-ΔΔCt^ method; RNAseq expression values are from DEseq normalized read counts. In all cases, expression was normalized to expression of WT grown in the dark (WT-D = 1). Error bars show standard error of the mean. For qPCR data, *t*-tests were performed to compare expression in WT-D to WT grown in 60 min R (WT-R), *phyA* mutants grown in the dark (*phyA*-D) to *phyA* mutants grown in 60 min of R (*phyA*-R), WT-D to *phyA*-D, and WT-R to *phyA*-R. Brackets indicate *p* ≤ 0.05 in that sample comparison. For RNAseq data, brackets indicate significant differential expression found in RNAseq analysis in that sample comparison. Note that while the expression patterns are similar between experiments, the magnitude of expression differs in some comparisons and the y-axis was adjusted accordingly.

Figure S3 Differential expression of translation related transcripts and proteins from enriched molecular function GO categories. DEPs and DETs with annotations related to translation were enriched in *phyA* to WT comparisons. Normalized read counts (DETs) or normalized spot volumes (DEPs) were *Z*-score normalized to a color scale where white represents averaged expression across genotype/conditions.

Figure S4 Free ATP content remains constant after 60 min R and between WT and *phyA* mutants. ATP content was measured using luminescence and quantified as nmol per gram fresh weight. Nine biological replicates of pooled seedlings were use for each genotype/condition. An ANOVA was performed showing no significant difference between ATP levels in any genotype/condition.

Figure S5 Greenyellow, red, and yellow networks show complex regulation by phyA and contain genes related to central metabolism. The greenyellow network **(A)** shows laccases (involved, e.g., in cell wall lignification) highlighted in blue. The red network **(B)** shows *SWEETs* highlighted in yellow. The yellow network **(C)** shows photosynthesis genes highlighted in blue. Gene names and IDs, network membership, and highlighted genes can be found in Supplementary Table S7.

Figure S6 Green and tan networks show severe mis-regulation in *phyA* mutants and contain translation and photosynthesis related genes (highlighted in yellow), respectively. Gene names and IDs, network membership, and highlighted genes can be found in Supplementary Table S7.

Figure S7 Blue and brown networks show regulation by phyA and other phytochromes that is light responsive. The blue network **(A)** is enriched in transcription factors which are highlighted in yellow and kinases highlighted in red. The brown network **(B)** is enriched in chromatin remodelers highlighted in yellow and protein modifiers highlighted in red. Gene names and IDs, network membership, and highlighted genes can be found in Supplementary Table S7.

Figure S8 PhyA reduces shoot elongation in dark grown seedlings regardless of light condition during sterilization/imbibition in 15-day-old seedlings. Seedlings of WT and *phyA* mutants were germinated in light-excluding boxes for 2 days and checked for germination. Only synchronized seedlings with roughly 2 mm long protruding radicles were used for subsequent experiments. A subset of seedlings was removed on days 5, 10, and 15, scanned, and their shoot length measured using ImageJ. To determine if light exposure during seedling sterilization and imbibition prior to sowing the seeds in the boxes made a difference, seed batches were either sterilized/imbibed, and sown in green safe light (“total dark”) or ambient lab light (“ambient”). Subsequently, both batches were grown in complete darkness without any light treatment for germination. Two-way ANOVAs were conducted in R for data at day 5, day 10, and day 15. *P*-values are shown and more information is available in Supplementary Tables S9, S10. 10–12 seedlings were collected for each genotype, condition, and time point for six biological replicates (total for all three time points *N* = 838). Condition (ambient light or total darkness) had no significant effect at day 5, and day 15. Condition showed a statistically significant effect at day 10, which might be due to the fact that at that time ambient *phyA* and total dark WT were statistically significantly different from each other. This difference is difficult to explain because even if ambient light had had an effect on growth it would be expected to decrease, not increase, hypocotyl length. Genotype had a significant effect 10 and 15 days post imbibition with *phyA* seedlings being significantly taller (see Figure 5).

Table S1 All differentially expressed proteins with average normalized spot volume and standard error for each genotype/condition across biological replicates.

Table S2 All differentially expressed transcripts with average normalized read count and standard error for each genotype/condition across biological replicates. Normalized read counts are from DESeq.

Table S3 Genes found to be differentialy expressed after 60 min of red light in both *A. thaliana* (Tepperman et al., 2006) and *S. lycopersicum*. Only genes with reciprocal best BLAST hit orthologs between *A. thaliana* and *S. lycopersicum* were compared.

Table S4 Fold induction and fold repression values for WT and phyA mutants in *A. thaliana* (Tepperman et al., 2006) and *S. lycopersicum*. Gray highlighted genes show matching regulation in *A. thaliana* and *S. lycopersicum*. Red highlighted genes show opposite regulation in *A. thaliana* and *S. lycopersicum*.

Table S5 All significant GO results for each comparison in DETs and DEPs (p < 0.05) using the weighted model in topGO and Fisher’s Exact Test.

Table S6 DETs and DEPs annotated as being involved in glycolysis, gluconeogenesis, the TCA cycle, the glyoxylate cycle, or beta oxidation. See Figure 2.

Table S7 The top 8000 most variable DETs and their co-expression modules. Module Membership p-value is the p-value from the Pearson correlation of the expression of the gene with the eigengene of that network.

Table S8 All significant GO results for each co-expression network (genes from module with topological overlap > 0.2).

Table S9 Raw data for dark growth analysis shown in Figure 5 and Supplementary Figure S8.

Table S10 Two-way ANOVA results from Supplementary Figure S8 and *t*-test results from Figure 5.

Table S11 Chlorophyll measurements per mg fresh wait in dark grown WT and phyA seedlings. Total Dark samples were sterilized and imbibed in the dark. Light exposed samples were sterilized and imbibed in the light.

Table S12 Raw data for sucrose response analysis. See Figure 6.

Table S13 Three-way ANOVA results from Figure 6.

Table S14 Primers used for qPCR analysis in Supplementary Figure S2.

MATERIAL Edge files for input to Cytoscape for each of the color modules. - Cytoscape input file for grey module - Cytoscape input file for turquoise module - Cytoscape input file for purple module - Cytoscape input file for green module - Cytoscape input file for magenta module - Cytoscape input file for black module - Cytoscape input file for brown module - Cytoscape input file for pink module - Cytoscape input file for blue module - Cytoscape input file for yellow module - Cytoscape input file for red module - Cytoscape input file for greenyellow module - Cytoscape input file for tan module
