# Supplementary figures and images for: Phytochrome A Regulates Carbon Flux in Dark Grown Tomato Seedlings
Source: Front Plant Sci. 2019 Feb 27;10:152. doi: 10.3389/fpls.2019.00152 (PMC6400891; doi:10.3389/fpls.2019.00152)

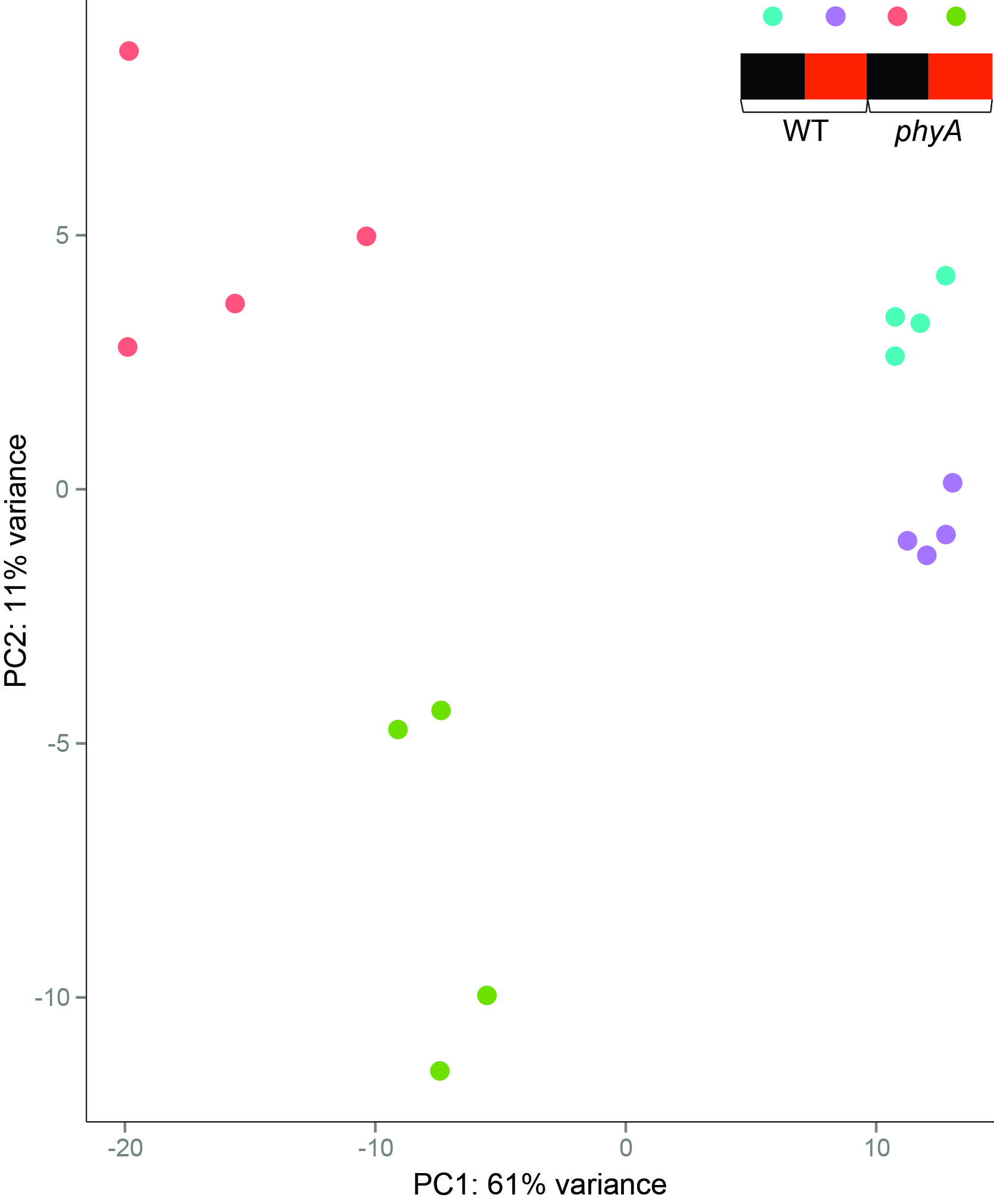

Supplement: Figure S1 — Principal components analysis of RNAseq data shows clustering of biological replicates. RNAseq data from DESeq analysis of four biological replicates of each genotype and condition combination was analyzed by principal components analysis. X-axis shows principal component 1 (PC1) and Y-axis shows principal component 2 (PC2). Dots represent individual biological replicates from the sample groups WT in dark (blue), WT in R (purple), phyA in dark (pink), phyA in R (green). [file Image_1.JPEG]

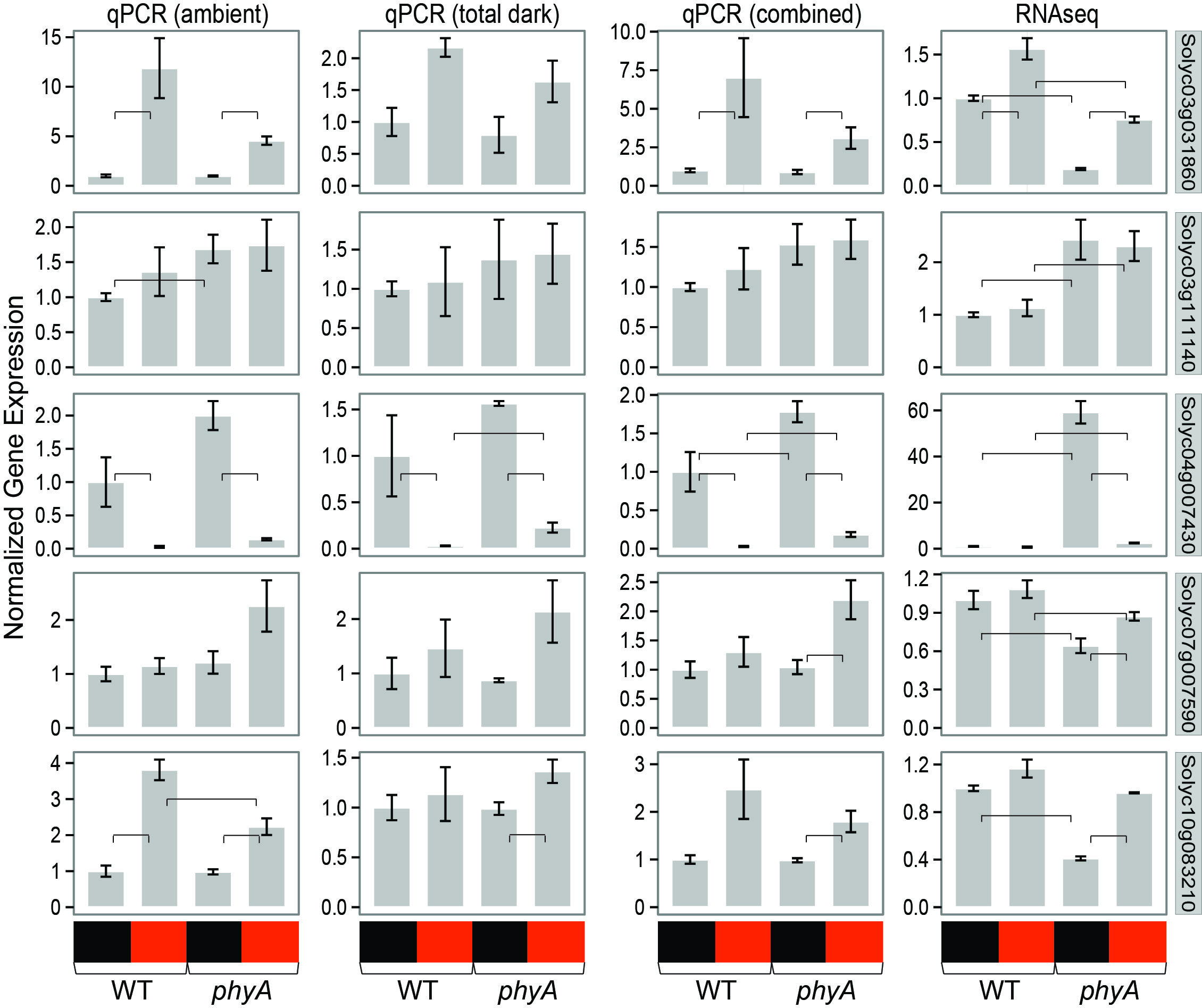

Supplement: Figure S2 — QPCR validation of expression patterns of five genes from RNAseq analysis. Normalized gene expression of five genes from two qPCR experiments (first three columns) and RNAseq experiment (last column) is shown. QPCR experiments were performed to replicate RNAseq experiment, one with seed sterilization in ambient lab light like done for RNAseq (ambient) and the other with seed sterilization in dark with green safe light (total dark) as a control. QPCR expression was calculated with the 2-ΔΔCt method; RNAseq expression values are from DEseq normalized read counts. In all cases, expression was normalized to expression of WT grown in the dark (WT-D = 1). Error bars show standard error of the mean. For qPCR data, t-tests were performed to compare expression in WT-D to WT grown in 60 min R (WT-R), phyA mutants grown in the dark (phyA-D) to phyA mutants grown in 60 min of R (phyA-R), WT-D to phyA-D, and WT-R to phyA-R. Brackets indicate p ≤ 0.05 in that sample comparison. For RNAseq data, brackets indicate significant differential expression found in RNAseq analysis in that sample comparison. Note that while the expression patterns are similar between experiments, the magnitude of expression differs in some comparisons and the y-axis was adjusted accordingly. [file Image_2.JPEG]

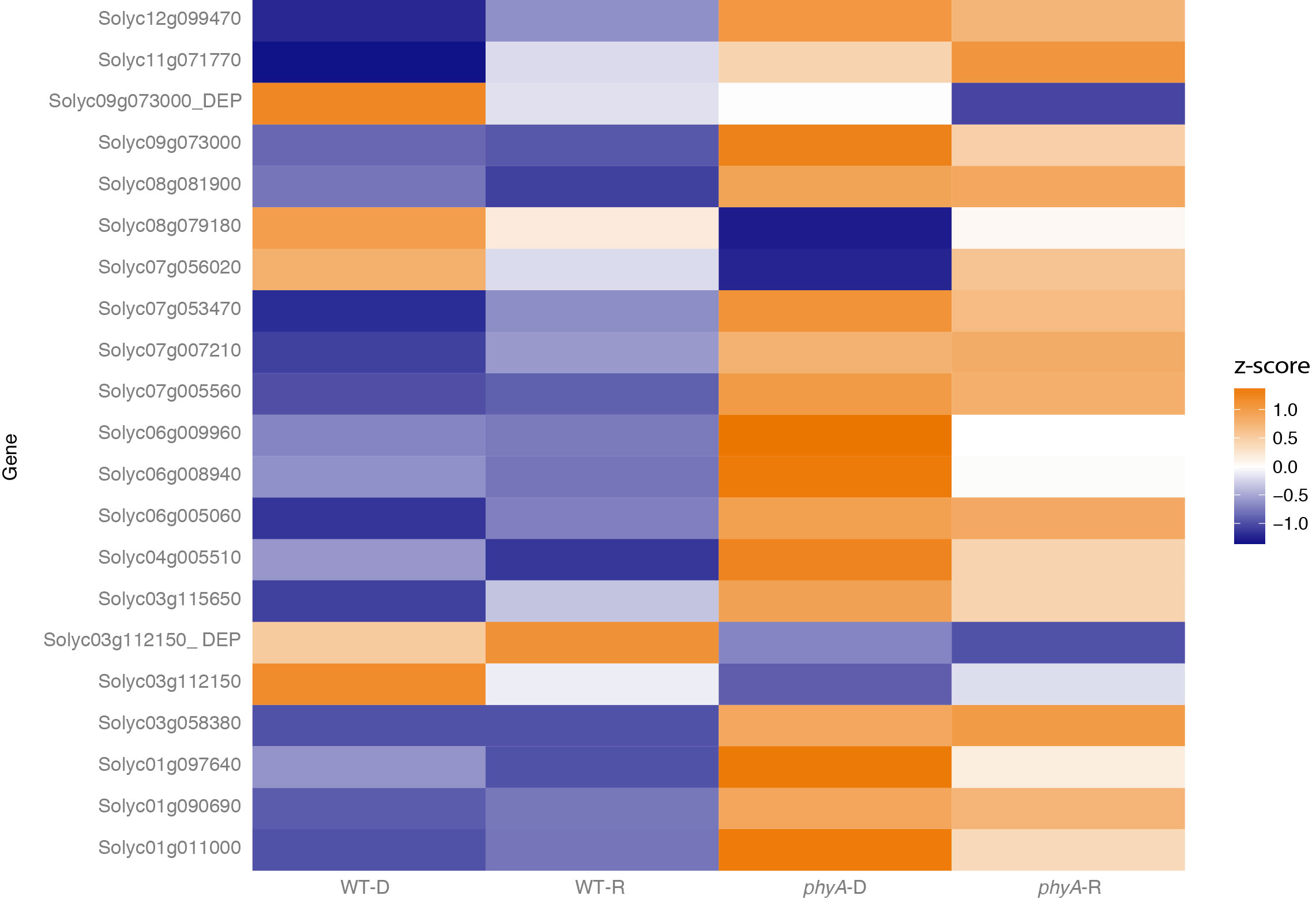

Supplement: Figure S3 — Differential expression of translation related transcripts and proteins from enriched molecular function GO categories. DEPs and DETs with annotations related to translation were enriched in phyA to WT comparisons. Normalized read counts (DETs) or normalized spot volumes (DEPs) were Z-score normalized to a color scale where white represents averaged expression across genotype/conditions. [file Image_3.JPEG]

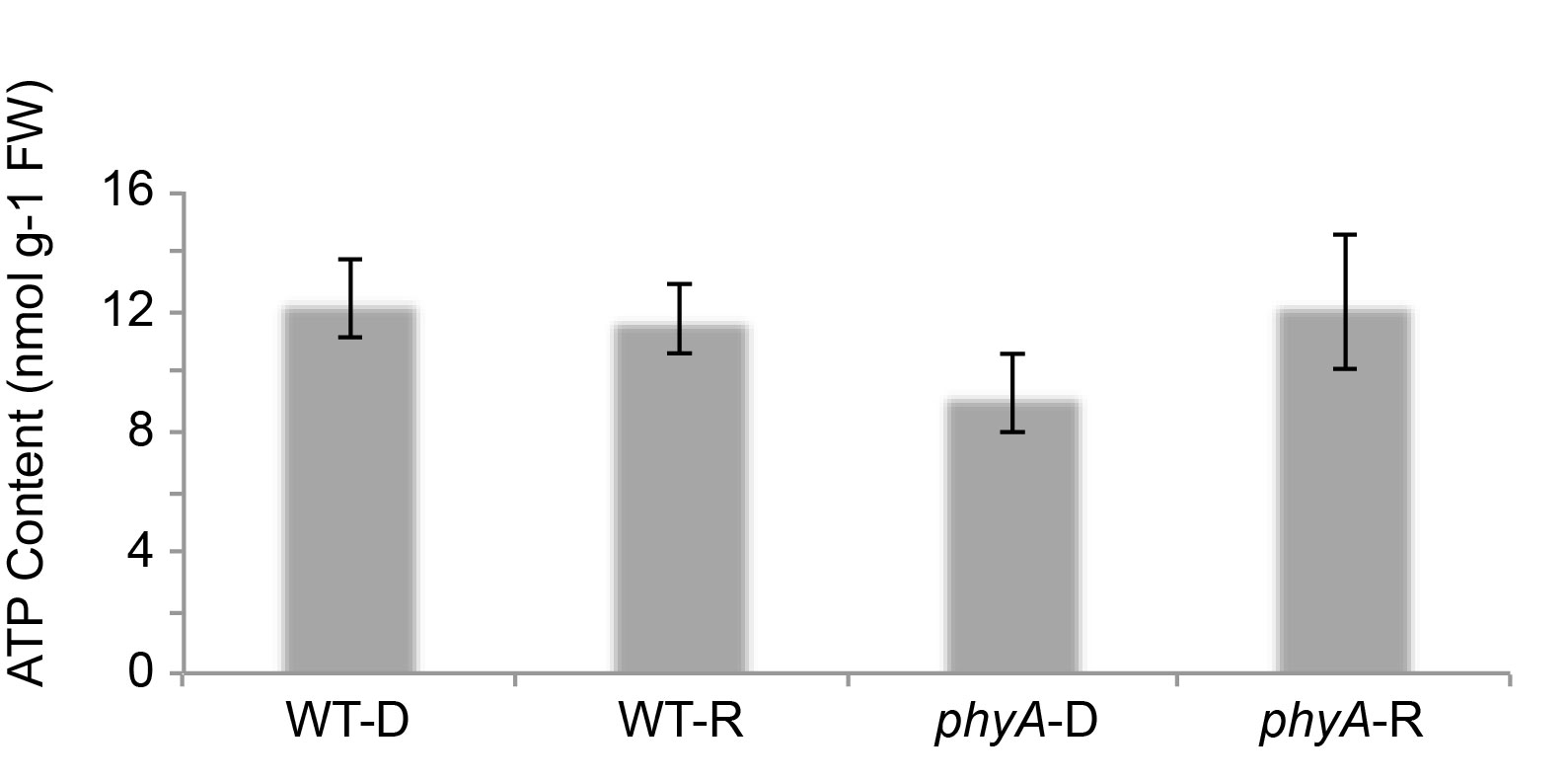

Supplement: Figure S4 — Free ATP content remains constant after 60 min R and between WT and phyA mutants. ATP content was measured using luminescence and quantified as nmol per gram fresh weight. Nine biological replicates of pooled seedlings were use for each genotype/condition. An ANOVA was performed showing no significant difference between ATP levels in any genotype/condition. [file Image_4.JPEG]

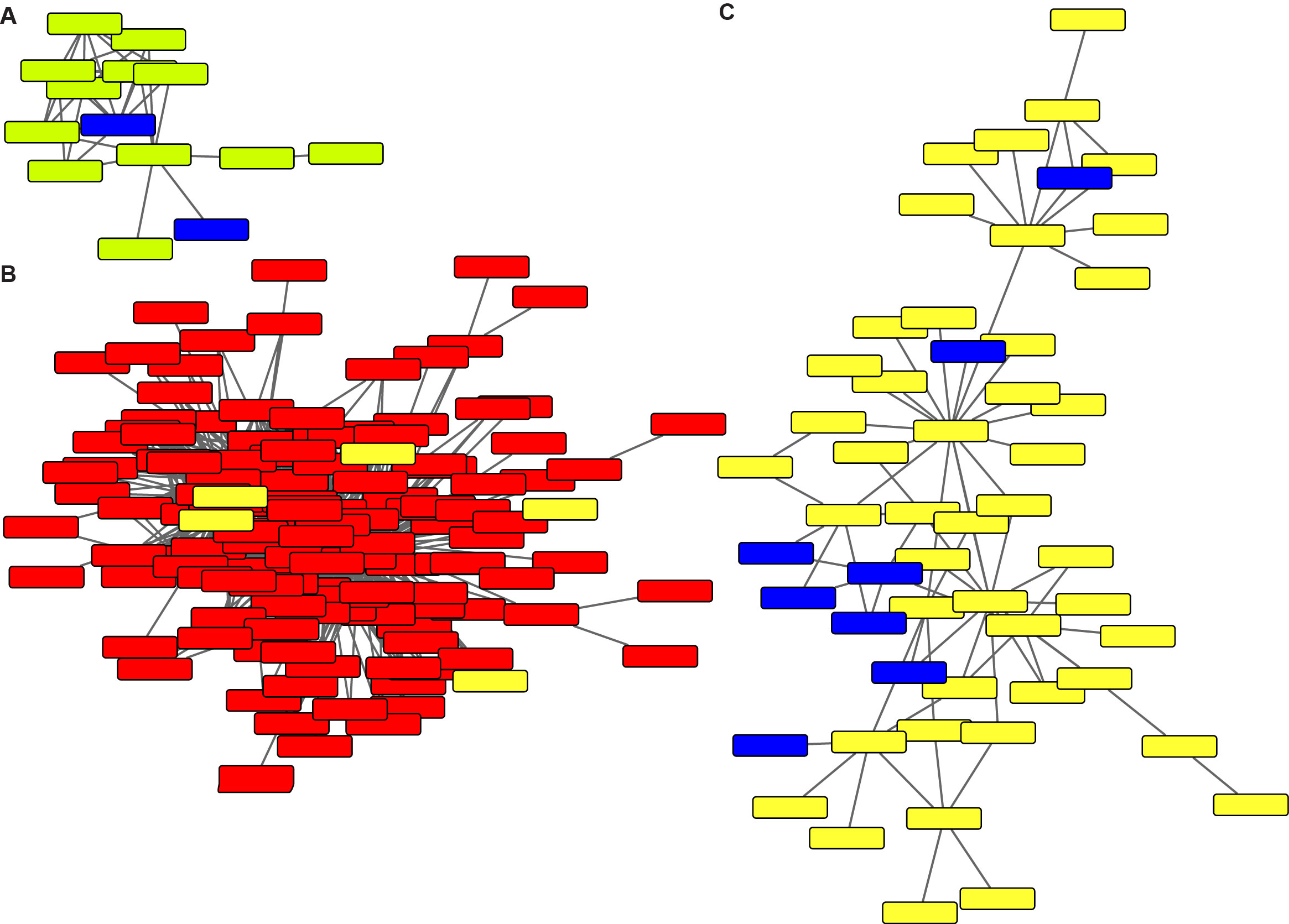

Supplement: Figure S5 — Greenyellow, red, and yellow networks show complex regulation by phyA and contain genes related to central metabolism. The greenyellow network (A) shows laccases (involved, e.g., in cell wall lignification) highlighted in blue. The red network (B) shows SWEETs highlighted in yellow. The yellow network (C) shows photosynthesis genes highlighted in blue. Gene names and IDs, network membership, and highlighted genes can be found in Supplementary Table S7. [file Image_5.jpeg]

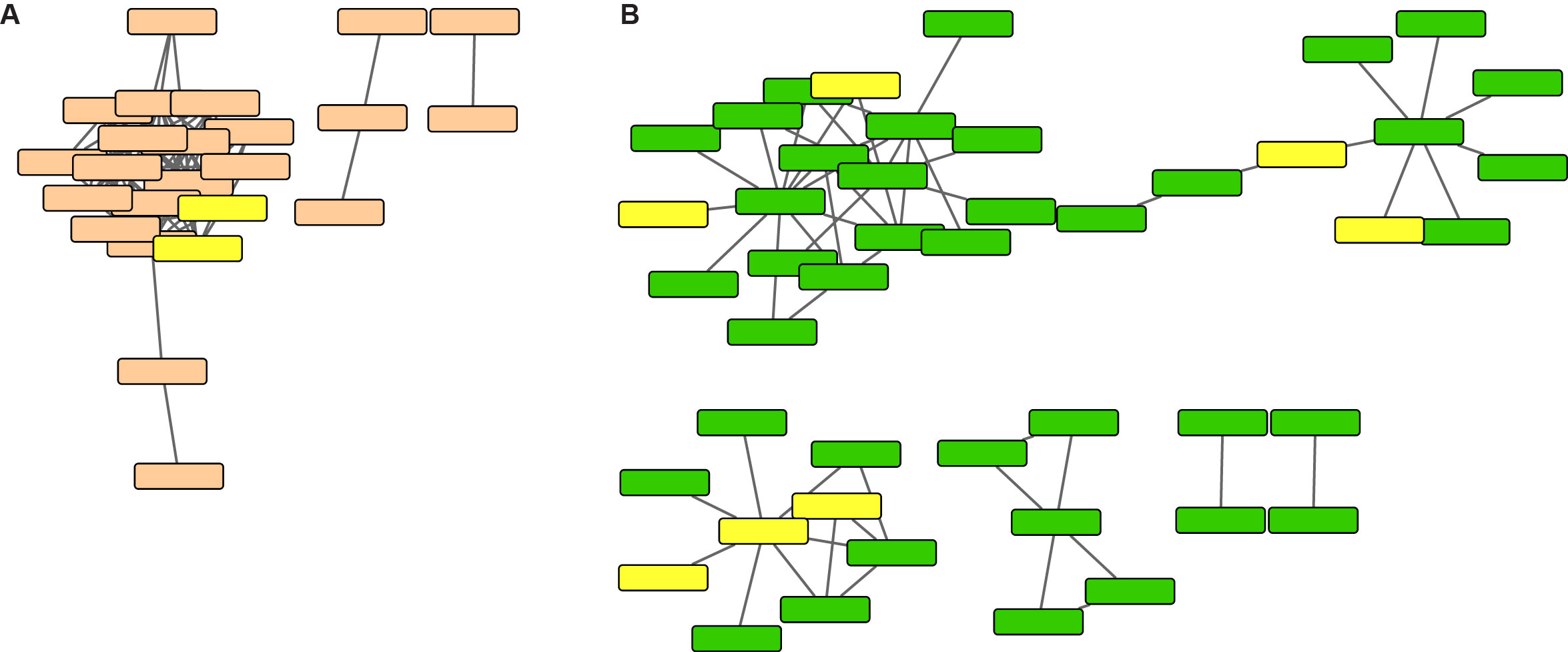

Supplement: Figure S6 — Green and tan networks show severe mis-regulation in phyA mutants and contain translation and photosynthesis related genes (highlighted in yellow), respectively. Gene names and IDs, network membership, and highlighted genes can be found in Supplementary Table S7. [file Image_6.JPEG]

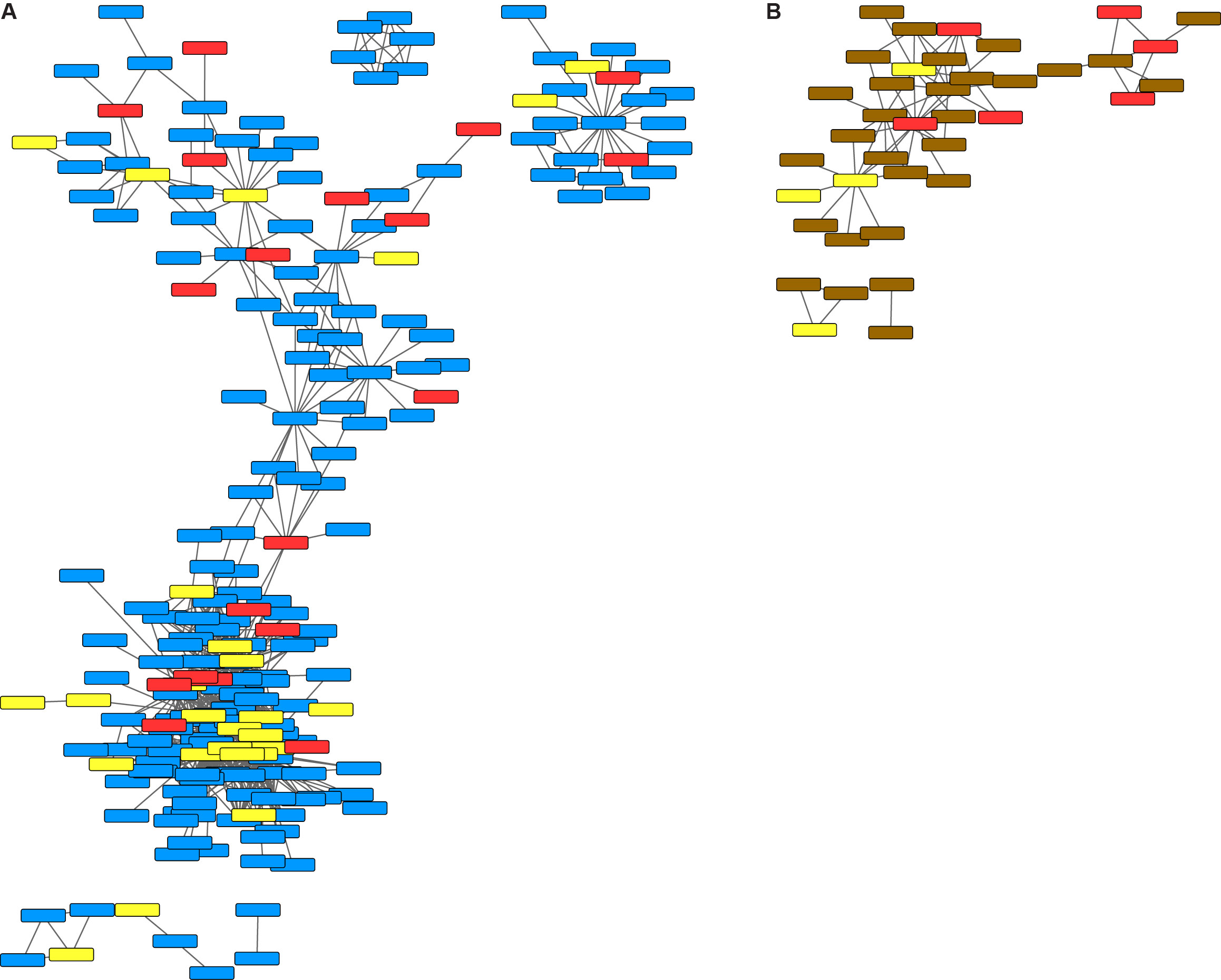

Supplement: Figure S7 — Blue and brown networks show regulation by phyA and other phytochromes that is light responsive. The blue network (A) is enriched in transcription factors which are highlighted in yellow and kinases highlighted in red. The brown network (B) is enriched in chromatin remodelers highlighted in yellow and protein modifiers highlighted in red. Gene names and IDs, network membership, and highlighted genes can be found in Supplementary Table S7. [file Image_7.JPEG]

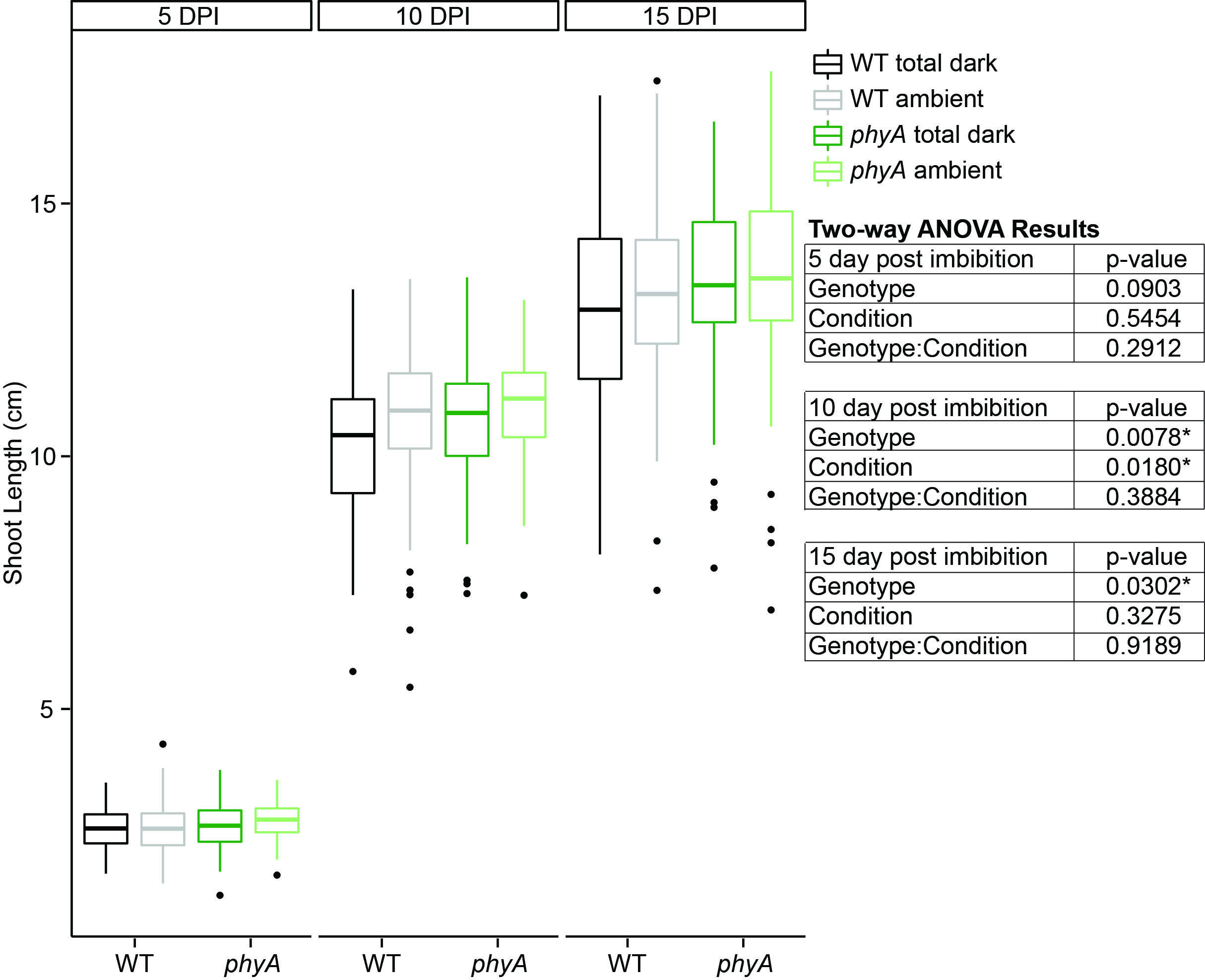

Supplement: Figure S8 — PhyA reduces shoot elongation in dark grown seedlings regardless of light condition during sterilization/imbibition in 15-day-old seedlings. Seedlings of WT and phyA mutants were germinated in light-excluding boxes for 2 days and checked for germination. Only synchronized seedlings with roughly 2 mm long protruding radicles were used for subsequent experiments. A subset of seedlings was removed on days 5, 10, and 15, scanned, and their shoot length measured using ImageJ. To determine if light exposure during seedling sterilization and imbibition prior to sowing the seeds in the boxes made a difference, seed batches were either sterilized/imbibed, and sown in green safe light (“total dark”) or ambient lab light (“ambient”). Subsequently, both batches were grown in complete darkness without any light treatment for germination. Two-way ANOVAs were conducted in R for data at day 5, day 10, and day 15. P-values are shown and more information is available in Supplementary Tables S9, S10. 10–12 seedlings were collected for each genotype, condition, and time point for six biological replicates (total for all three time points N = 838). Condition (ambient light or total darkness) had no significant effect at day 5, and day 15. Condition showed a statistically significant effect at day 10, which might be due to the fact that at that time ambient phyA and total dark WT were statistically significantly different from each other. This difference is difficult to explain because even if ambient light had had an effect on growth it would be expected to decrease, not increase, hypocotyl length. Genotype had a significant effect 10 and 15 days post imbibition with phyA seedlings being significantly taller (see Figure 5). [file Image_8.JPEG]
